# Propofol Alleviates Anxiety‐Like Behaviors Associated with Pain by Inhibiting the Hyperactivity of PVN^CRH^ Neurons via GABA_A_ Receptor *β*3 Subunits

**DOI:** 10.1002/advs.202309059

**Published:** 2024-04-19

**Authors:** Le Yu, Xiaona Zhu, Kang Peng, Huimin Qin, Kexin Yang, Fang Cai, Ji Hu, Ye Zhang

**Affiliations:** ^1^ Department of Anesthesiology The Second Affiliated Hospital of Anhui Medical University Hefei 230601 China; ^2^ Key Laboratory of Anesthesiology and Perioperative Medicine of Anhui Higher Education Institutes Anhui Medical University Hefei 230032 China; ^3^ School of Life Science and Technology ShanghaiTech University Shanghai 201210 China

**Keywords:** anxiety, corticotropin‐releasing hormone, excitatory‐inhibitory balance, GABA_A_ receptor, paraventricular nucleus, propofol

## Abstract

Pain, a comorbidity of anxiety disorders, causes substantial clinical, social, and economic burdens. Emerging evidence suggests that propofol, the most commonly used general anesthetic, may regulate psychological disorders; however, its role in pain‐associated anxiety is not yet described. This study investigates the therapeutic potential of a single dose of propofol (100 mg kg^−1^) in alleviating pain‐associated anxiety and examines the underlying neural mechanisms. In acute and chronic pain models, propofol decreased anxiety‐like behaviors in the elevated plus maze (EPM) and open field (OF) tests. Propofol also reduced the serum levels of stress‐related hormones including corticosterone, corticotropin‐releasing hormone (CRH), and norepinephrine. Fiber photometry recordings indicated that the calcium signaling activity of CRH neurons in the paraventricular nucleus (PVN^CRH^) is reduced after propofol treatment. Interestingly, artificially activating PVN^CRH^ neurons through chemogenetics interfered with the anxiety‐reducing effects of propofol. Electrophysiological recordings indicated that propofol decreases the activity of PVN^CRH^ neurons by increasing spontaneous inhibitory postsynaptic currents (sIPSCs). Further, reducing the levels of *γ*‐aminobutyric acid type A receptor *β*3 (GABA_A_ *β*3) subunits in PVN^CRH^ neurons diminished the anxiety‐relieving effects of propofol. In conclusion, this study provides a mechanistic and preclinical rationale to treat pain‐associated anxiety‐like behaviors using a single dose of propofol.

## Graphical abstract

A single dose of propofol alleviates anxiety‐like behaviors associated with pain. This therapeutic effect is attributed to its action on GABA_A_ *β*3 subunits, which restores the balance between excitation and inhibition and subsequently suppresses the excessive activation of PVN^CRH^ neurons in pain. These findings provide insights into the potential application of propofol for treating psychiatric and emotional disorders.

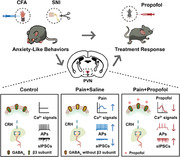

## 1 Introduction

Pain, especially chronic pain, frequently leads to maladaptive psychological responses, including anxiety.^[^ ^1^ ^]^ In particular, anxiety can exacerbate pain intensity and duration, potentially leading to a vicious cycle of pain and anxiety, resulting in a considerable burden on patients, families, and society.^[^ ^2^ ^]^ However, in a clinical setting, the management of physical pain is the primary focus, while the treatment of pain‐associated emotional reactions, such as anxiety associated with pain, is often neglected. Effective medications and interventions to treat anxiety associated with pain could reduce the incidence of subsequent severe emotional disorders and improve patients quality of life.

Propofol, a drug that won the Lasker–DeBakey Clinical Medical Research Award, exerts general anesthetic effects via the *γ*‐aminobutyric acid type A receptor (GABA_A_).^[^ ^3^ ^]^ In clinical practice, patients receiving propofol anesthesia commonly experience intense and enduring sensations of euphoria.^[^ ^4^ ^]^ This implies that propofol is a promising drug for the treatment of mood disorders. A previous study reported that propofol could reverse anhedonia by inhibiting dopamine transporters.^[^ ^4a^ ^]^ Additionally, clinical studies have suggested that propofol can alleviate perioperative anxiety by inhibiting the activation of the hypothalamic–pituitary–adrenal (HPA) axis to maintain the homeostasis.^[^ ^5^ ^]^ However, whether propofol can alleviate pain‐associated anxiety responses has not yet been determined, and its neural mechanisms and molecular targets have not yet been investigated.

Corticotropin‐releasing hormone (CRH) neurons located in the paraventricular nucleus (PVN) of the hypothalamus are crucial for controlling the HPA stress‐response axis.^[^ ^6^ ^]^ Stress activates the PVN^CRH^ neurons to release CRH. Subsequently, CRH enters the pituitary gland and initiates a cascading response, ultimately leading to the release of a large amount of corticosterone (CORT), which disrupts homeostasis and can eventually lead to mental health issues.^[^ ^7^ ^]^ The activation of PVN^CRH^ neurons is associated with an increased risk of anxiety. We have previously indicated that stress can induce increased excitability of PVN^CRH^ neurons by disrupting synaptic homeostasis, resulting in anxiety‐like behaviors and a surge in stress‐related hormones in mice.^[^ ^8^ ^]^ Moreover, chemogenetic activation of PVN^CRH^ neurons promotes anxiety‐like behaviors. Pain induces increased c‐Fos expression in PVN^CRH^ neurons, providing evidence that PVN^CRH^ neurons rapidly respond to pain stimulation.^[^ ^9^ ^]^ Furthermore, a pilot in vitro electrophysiological investigation has indicated that propofol acts on GABA_A_ receptors in neurons within the PVN, resulting in enhanced inhibitory currents.^[^ ^10^ ^]^ Based on these studies, PVN^CRH^ neurons are likely to be crucial neural targets through which propofol may alleviate anxiety‐like behaviors associated with pain.

In this study, we investigated whether the acute administration of different doses of propofol could modulate the excitability of PVN^CRH^ neurons to regulate anxiety‐like behaviors associated with pain. Using acute and chronic pain models, we demonstrated that a single dose of propofol (100 mg kg^−1^) alleviated anxiety‐like behaviors and suppressed stress‐related hormone surges. Furthermore, our findings demonstrate that propofol effectively reduces the calcium signaling activity in PVN^CRH^ neurons. Local injection of propofol has been shown to alleviate anxiety‐like behaviors in mice with CFA‐induced pain. However, when we chemogenetically activated PVN^CRH^ neurons blocked the anxiety‐relieving effects of propofol. Additionally, we observed that the knockdown of *γ*‐aminobutyric acid type A receptor *β*3 (GABA_A_ *β*3) subunits in PVN^CRH^ neurons attenuated the therapeutic effects of propofol. These results establish a foundation for the potential use of propofol to treat anxiety‐like behaviors associated with pain.

## 2 Results

### 2.1 Propofol Alleviates Anxiety‐Like Behaviors and Endocrine Responses Associated with Pain

**Figure 1:**
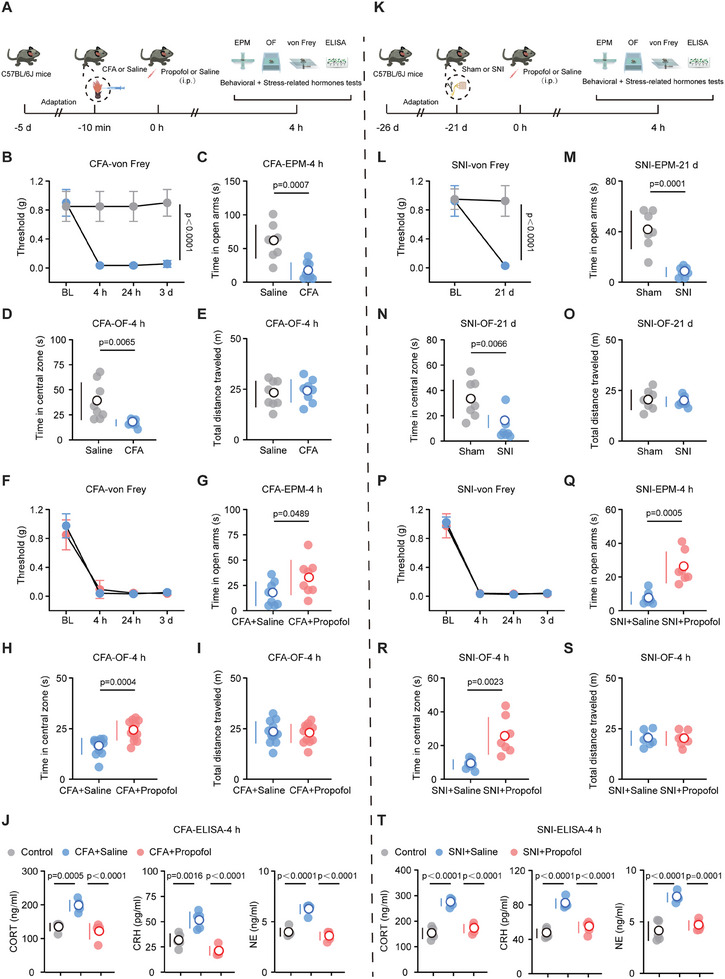
Propofol alleviates anxiety‐like behaviors and the stress‐related hormones surge associated with pain. A) Schematic of the injection of CFA and propofol along with behavioral and stress‐related hormones tests. B) Paw withdrawal threshold over time for saline and CFA groups (*n* = eight mice per group). C) Time in the open arms during the EPM test for saline and CFA groups at 4 h after saline or CFA injection (*n* = eight mice per group). D) Time in the central zone during the OF test for saline and CFA groups at 4 h after saline or CFA injection (*n* = eight mice per group). E) Total distance traveled during the OF test for saline and CFA groups at 4 h after saline or CFA injection (*n* = eight mice per group). F) Paw withdrawal threshold over time for CFA + saline and CFA + propofol (100 mg kg^−1^) groups at 4 h after drug injection (*n* = eight mice per group). G) Time in the open arms during the EPM test for CFA + saline and CFA + propofol (100 mg kg^−1^) groups at 4 h after drug injection (*n* = eight mice per group). H) Time in the central zone during the OF test for CFA + saline and CFA + propofol (100 mg kg^−1^) groups at 4 h after drug injection (*n* = twelve mice per group). I) Total distance traveled during the OF test in CFA + saline and CFA + propofol (100 mg kg^−1^) groups at 4 h after drug injection (*n* = twelve mice per group). J) Serum CORT concentrations (left), serum CRH concentrations (middle), and serum NE concentrations (right) for control, CFA + saline, and CFA + propofol (100 mg kg^−1^) groups at 4 h after drug injection (*n* = five mice per group). K) Schematic of the surgery of SNI and propofol along with behavioral and stress‐related hormones tests. L) Paw withdrawal threshold over time for sham and SNI groups (*n* = eight mice per group) M) Time in the open arms during the EPM test for sham and SNI groups at 21 days after surgery (*n* = seven mice per group). N) Time in the central zone during the OF test for sham and SNI groups at 21 days after surgery (*n* = seven mice per group). O) Total distance traveled during the OF test for sham and SNI groups at 21 days after surgery (*n* = seven mice per group). P) Paw withdrawal threshold over time for SNI + saline and SNI + propofol (100 mg kg^−1^) groups (*n* = eight mice per group). Q) Time in the open arms during the EPM test for SNI + saline and SNI + propofol (100 mg kg^−1^) groups at 4 h after drug injection (*n* = seven mice per group). R) Time in the central zone during the OF test for SNI + saline and SNI + propofol (100 mg kg^−1^) groups at 4 h after drug injection (*n* = seven mice per group). S) Total distance traveled during the OF test in SNI + saline and SNI + propofol (100 mg kg^−1^) groups at 4 h after drug injection (*n* = seven mice per group). T) Serum CORT concentrations (left), serum CRH concentrations (middle), and serum NE concentrations (right) for sham, SNI + saline, and SNI + propofol (100 mg kg^−1^) groups at 4 h after drug injection (*n* = five mice per group). Data are shown as the mean (white circles) ± SD (vertical lines) along with individual data points and were compared using two‐tailed, unpaired Student's t‐test (C–E, G–I, M–O and Q–S) or two‐way ANOVA followed by Tukey's multiple comparisons test (B, F, L and P) or one‐way ANOVA followed by Tukey's multiple comparisons test (J and T). CFA, complete Freund's adjuvant; SNI, spared nerve injury; EPM, elevated plus maze; OF, open field; ELISA, enzyme linked immunosorbent assay; CORT, corticosterone; CRH, corticotropin‐releasing hormone; NE, norepinephrine; BL, baseline; CFA + Saline, CFA‐injected mice exposed to saline; CFA + Propofol, CFA‐injected mice exposed to propofol; SNI + Saline, SNI mice exposed to saline; SNI + Propofol, SNI mice exposed to propofol.

To examine the therapeutic effects of propofol on acute pain‐associated anxiety, we injected 25 µL of CFA into the left hind paw of mice to establish a model of inflammatory pain (**Figure** **1**A).^[^ ^11^ ^]^ Four hours after the CFA injection, the mice displayed a significant reduction in their mechanical thresholds, as determined by von Frey tests (Figure 1B), and increased anxiety‐like behaviors (Figure 1C–E). Specifically, the CFA mice spent 70% less time in the open arms of the elevated plus maze (EPM) test (Figure 1C) and 50% less time in the central zone of the open field (OF) test (Figure 1D) compared to the saline‐treated mice; nevertheless, the total distance traveled did not change during the OF test between the CFA and the saline groups (Figure 1E). Additionally, time‐course experiments indicated that anxiety‐like behaviors and mechanical hyperalgesia persisted for up to 3 days after the CFA injection (Figure 1B; Figure S1, Supporting Information). These data, consistent with those of previous studies, confirm that pain induces anxiety‐like behaviors in mice.^[^ ^12^ ^]^

Patients who receive propofol anesthesia experience euphoria and relief from postoperative anxiety,^[^ ^4b^ ^]^ and a single dose of propofol (100 mg kg^−1^) does indeed have a therapeutic effect on stress‐induced anhedonia in mice.^[^ ^4a^ ^]^ Therefore, we were interested in whether a single dose of propofol (25, 50, or 100 mg kg^−1^) could reduce acute pain‐associated anxiety‐like behaviors. A single propofol dose of 100 mg kg^−1^ (but not 25 or 50 mg kg^−1^) administered to mice 10 min after the CFA injection alleviated anxiety‐like behaviors 4 h after treatment (Figure 1G–I; Figure S2, Supporting Information), although 100 mg kg^−1^ propofol did not affect mechanical hypersensitivity (Figure 1F). We recently demonstrated that propofol has long‐lasting anti‐anhedonic effects in mice. Therefore, we implemented the EPM, OF, and von Frey tests 24 h and 3 days after a single dose of propofol (100 mg kg^−1^). Strikingly, the therapeutic effects of propofol on acute pain‐associated anxiety‐like behaviors were observed for at least 3 days after treatment (Figure S3, Supporting Information). Furthermore, we ruled out the effects of the solvent intralipid (Figure S4, Supporting Information).

Furthermore, we investigated stress‐related hormones in the plasma of control, CFA+saline, and CFA+propofol mice. The CFA+saline mice exhibited increased levels of CORT, CRH, and norepinephrine (NE) compared to the control mice (Figure 1J). However, a single dose of propofol (100 mg kg^−1^) resulted in a significant decrease in these stress‐related hormones (Figure 1J), and this inhibitory effect lasted for at least 3 days (Figure S5, Supporting Information). Thus, at both the behavioral and hormonal levels, a single dose of propofol (100 mg kg^−1^) produces rapid and long‐lasting effects on anxiety‐like behaviors associated with acute pain in mice.

Next, we conducted similar experiments to investigate the effects of propofol on anxiety‐like behaviors and mechanical thresholds in the context of chronic pain (Figure 1K). First, we established a spared nerve injury (SNI) chronic pain model. Twenty‐one days after the SNI surgery, the mice displayed a significant reduction in their mechanical thresholds for allodynia (Figure 1L) and anxiety‐like behaviors (Figure 1M–O). Subsequently, we injected 100 mg kg^−1^ propofol to assess changes in anxiety‐like behaviors and mechanical thresholds in the SNI mice. Similar to the results observed in the CFA‐injected mice, propofol did not improve mechanical hyperalgesia in the SNI mice (Figure 1P). Behavioral results suggested that propofol alleviated anxiety‐like behaviors associated with chronic pain (Figure 1Q–S), and this therapeutic effect was observed at least 3 days after treatment (Figure S6, Supporting Information). Finally, we established that propofol effectively inhibited the increase in the serum levels of CORT, CRH, and NE induced by chronic pain in the SNI mice, and this effect lasted for at least 3 days after treatment (Figure 1T; Figure S7, Supporting Information).

Overall, our results suggest that propofol alleviates anxiety‐like behaviors and endocrine responses associated with acute and chronic pain without improving mechanical hyperalgesia.

### 2.2 Propofol Reduces Pain‐Induced Hyperactivity of PVN^CRH^ Neurons in Mice

**Figure 2:**
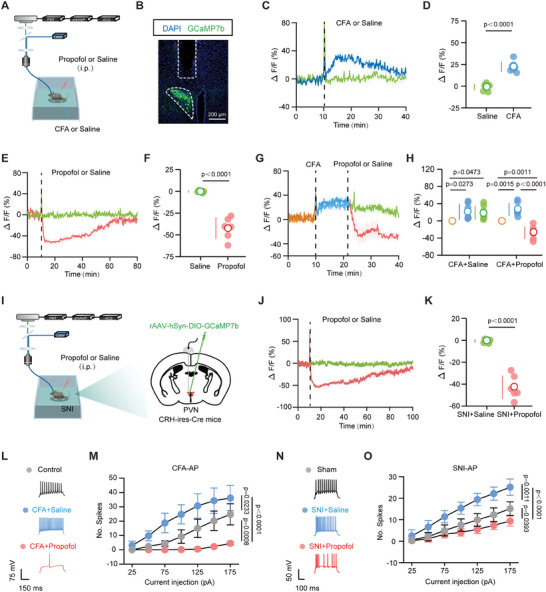
The response dynamics of PVN^CRH^ neurons to pain and propofol. A) Schematic of fiber implantation above PVN^CRH^ neurons expressing GCaMP7b in CRH‐ires‐Cre mice. B) Successful expression of GCaMP7b in PVN^CRH^ neurons. Scale bar, 200 µm. C) Calcium signaling as determined by GCaMP7b fluorescence in PVN^CRH^ neurons relative to the moment of intraplantar injection of saline or CFA (*n* = seven mice per group). D) Quantification of changes in GCaMP7b signals after administration of saline and CFA. E) Calcium signaling as determined by GCaMP7b fluorescence in PVN^CRH^ neurons relative to the moment of administration of saline or propofol (100 mg kg^−1^) (*n* = six mice per group). F) Quantification of changes in GCaMP7b signals after administration of saline and propofol (100 mg kg^−1^). G) GCaMP7b signals from PVN^CRH^ neurons over three periods: the baseline value before treatment, after CFA injection, and after propofol or saline injection (*n* = seven mice per group). H) Quantification of changes in GCaMP7b signals during these three periods. I) Schematic of fiber implantation above PVN^CRH^ neurons expressing GCaMP7b in CRH‐ires‐Cre mice. J) Calcium signaling as determined by GCaMP7b fluorescence in PVN^CRH^ neurons relative to the moment of administration of saline or propofol in SNI mice (100 mg kg^−1^) (*n* = six mice per group). K) Quantification of changes in GCaMP7b signals after administration of saline and propofol (100 mg kg^−1^) in SNI mice. L) Representative traces of the action potentials recorded from PVN^CRH^ neurons with 125 pA current injection from control, CFA + saline, and CFA+propofol groups. Scale bars, 75 mV, 150 ms. M) Relationship between current injection and the number of elicited action potential spikes in control, CFA + saline, and CFA + propofol groups (*n* = eight neurons in four mice for each group). N) Representative traces of the action potentials recorded from PVN^CRH^ neurons with 150 pA current injection from sham, SNI + saline, and SNI + propofol groups. Scale bars, 50 mV, 100 ms. O) Relationship between current injection and the number of elicited action potential spikes in sham, SNI + saline, and SNI + propofol groups (*n* = eight neurons in four mice for each group). Data are shown as the mean (white circles) ± SD (vertical lines) along with individual data points and were compared using two‐tailed, unpaired Student's *t*‐test (D, F, and K) or one‐way ANOVA followed by Tukey's multiple comparisons test (H) or two‐way ANOVA followed by Tukey's multiple comparisons test (M and O). CFA, complete Freund's adjuvant; SNI, spared nerve injury; PVN, paraventricular nucleus; CRH, corticotropin‐releasing hormone; CFA + Saline, CFA‐injected mice exposed to saline; CFA + Propofol, CFA‐injected mice exposed to propofol; SNI + Saline, SNI mice exposed to saline; SNI + Propofol, SNI mice exposed to propofol.

PVN^CRH^ neurons trigger hormonal cascades along the HPA axis and coordinate stress‐related behaviors.^[^ ^6^, ^7^, ^13^ ^]^ To quantify the physiological activity of PVN^CRH^ neurons during pain and propofol administration, we used fiber photometry to monitor calcium signal alterations in real‐time in PVN^CRH^ neurons of CRH‐ires‐Cre mice that had received an injection of the rAAV‐hSyn‐DIO‐GCaMP7b vector in the PVN (**Figure** **2**A,B).

First, we examined the effect of CFA‐induced pain on calcium signaling in PVN^CRH^ neurons, which exhibited increased PVN^CRH^ neuronal activity following the CFA injection (Figure 2C,D). Subsequently, we measured the response dynamics of the PVN^CRH^ neurons to different doses of propofol (25, 50, and 100 mg kg^−1^) in naïve CRH‐ires‐Cre mice. A single dose of 100 mg kg^−1^ propofol suppressed the calcium signals of PVN^CRH^ neurons, and this effect persisted for up to 80–90 min after drug administration (Figure 2E,F). At doses of 25 and 50 mg kg^−1^, propofol inhibited the activity of PVN neurons, and this inhibitory effect lasted for ≈40–60 min (Figure S8, Supporting Information). To determine the effect of propofol on pain‐induced activation of PVN^CRH^ neurons, mice were administered propofol 10 min after the CFA injection. The results indicated that propofol promptly reversed the neuronal activation induced by the CFA injection (Figure 2G,H). Furthermore, we recorded the effects of propofol on calcium activity in PVN^CRH^ neurons in the SNI mouse model of chronic pain (Figure 2I). We established that propofol (100 mg kg^−1^) decreased PVN^CRH^ neuronal activity in the SNI mice (Figure 2J,K).

Consistent with fiber photometry recordings, in vitro electrophysiological recordings revealed that propofol inhibited the increase in pain‐induced action potentials (APs). We recorded the activity of CRH neurons in PVN slices from the control, CFA+saline, and CFA+propofol mice. APs were evoked by 25‐pA step depolarizing current pulses ranging from −25 to 200 pA. In the CFA+saline mice, the PVN^CRH^ neurons exhibited increased neuronal excitability, which was reversed by propofol treatment (Figure 2L,M). Additionally, we recorded the activity of CRH neurons in PVN slices from the sham, SNI+saline, and SNI+propofol mice. PVN^CRH^ neurons in the SNI+saline mice also exhibited increased excitability compared to sham mice, whereas propofol inhibited neuronal firing (Figure 2N,O).

These results indicate that propofol decreases the hyperactivation of PVN^CRH^ neurons induced by pain.

### 2.3 Inhibiting PVN^CRH^ Neurons Alleviates Pain‐Associated Anxiety‐Like Behaviors

**Figure 3:**
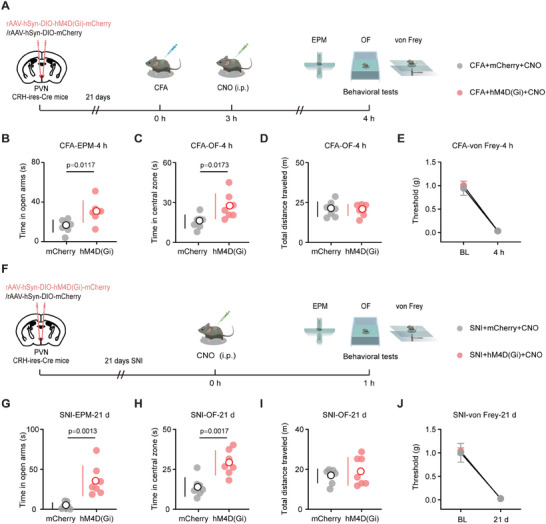
Inhibiting PVN^CRH^ neurons alleviates anxiety‐like behaviors associated with pain. A) Schematic of viral injection (mCherry or hM4D(Gi)) along with behavioral tests in CFA mice. B) Time in the open arms during the EPM test for mCherry and hM4D(Gi) groups in CFA mice (*n* = seven mice per group). C) Time in the central zone during the OF test for mCherry and hM4D(Gi) groups in CFA mice (*n* = seven mice per group). D) Total distance traveled during the OF test for mCherry and hM4D(Gi) groups in CFA mice (*n* = seven mice per group). (E) Paw withdrawal threshold for mCherry and hM4D(Gi) groups in CFA mice (*n* = seven mice per group). F) Schematic of viral injection (mCherry or hM4D(Gi)) along with behavioral tests in SNI mice. G) Time in the open arms during the EPM test for mCherry and hM4D(Gi) groups in SNI mice (*n* = seven mice per group). H) Time in the central zone during the OF test for mCherry and hM4D(Gi) groups in SNI mice (*n* = seven mice per group). I) Total distance traveled during the OF test for mCherry and hM4D(Gi) groups in SNI mice (*n* = seven mice per group). J) Paw withdrawal threshold for mCherry and hM4D(Gi) groups in SNI mice (*n* = seven mice per group). Data are shown as the mean (white circles) ± SD (vertical lines) along with individual data points and were compared using two‐tailed, unpaired Student's *t*‐test (B–D and G–I) or two‐way ANOVA followed by Tukey's multiple comparisons test (E and J). CFA, complete Freund's adjuvant; SNI, spared nerve injury; EPM, elevated plus maze; OF, open field; PVN, paraventricular nucleus; CRH, corticotropin‐releasing hormone; BL, baseline; CNO, Clozapine *N*‐oxide.

Given the hyperactivity of PVN^CRH^ neurons in response to anxiety‐like behaviors induced by pain, we assessed whether inhibiting PVN^CRH^ neurons could reverse anxiety‐like behaviors caused by pain. We administered rAAV‐hSyn‐DIO‐hM4D(Gi)‐mCherry (hM4D(Gi) group) or rAAV‐hSyn‐DIO‐mCherry (mCherry group) bilaterally into the PVN of CFA mice (**Figure** **3**A). The perfusion of brain slices with clozapine *N*‐oxide (CNO) decreased the firing rate of PVN^CRH^ neurons (Figure S9, Supporting Information). Compared to mice in the mCherry group, mice in the hM4D(Gi) group spent more time in the open arms of the EPM test (Figure 3B) and in the central zone of the OF test (Figure 3C), without exhibiting any effects on the total distance traveled (Figure 3D) or paw withdrawal threshold (Figure 3E), implying that chemogenetic inhibition of PVN^CRH^ neurons rescued anxiety‐like behaviors in the CFA‐4h mice. Similar results were observed in the SNI mice (Figure 3F–J).

### 2.4 Propofol Rapidly Attenuates Anxiety‐Like Behaviors Through the Inhibition of PVN^CRH^ Neurons

**Figure 4:**
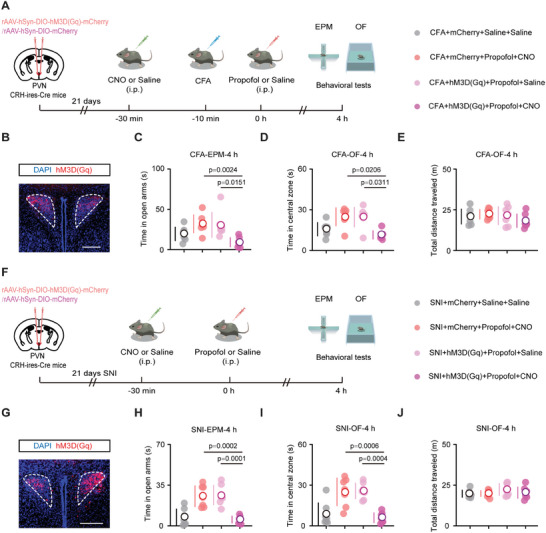
Inhibiting PVN^CRH^ neurons alleviates anxiety‐like behaviors associated with pain. A) Schematic of viral injection (mCherry or hM3D(Gq)) along with behavioral tests in CFA mice. B) Successful expression of hM3D(Gq) in PVN^CRH^ neurons in CFA mice. Scale bar, 200 µm. C) Time in the open arms during the EPM test for mCherry+saline+saline, mCherry+propofol+CNO, hM3D(Gq)+propofol+saline, and hM3D(Gq)+propofol+CNO groups in CFA mice at 4 h after treatment (*n* = seven mice per group). D) Time in the central zone during the OF test for mCherry+saline+saline, mCherry+propofol+CNO, hM3D(Gq)+propofol+saline, and hM3D(Gq)+propofol+CNO groups in CFA mice at 4 h after treatment (*n* = seven mice per group). E) Total distance traveled during the OF test for mCherry+saline+saline, mCherry+propofol+CNO, hM3D(Gq)+propofol+saline, and hM3D(Gq)+propofol+CNO groups in CFA mice at 4 h after treatment (*n* = seven mice per group). F) Schematic of viral injection (mCherry or hM3D(Gq)) along with behavioral tests in SNI mice. G) Successful expression of hM3D(Gq) in PVN^CRH^ neurons in SNI mice. Scale bar, 200 µm. H) Time in the open arms during the EPM test for mCherry+saline+saline, mCherry+propofol+CNO, hM3D(Gq)+propofol+saline, and hM3D(Gq)+propofol+CNO groups in SNI mice at 4 h after treatment (*n* = seven mice per group). I) Time in the central zone during the OF test for mCherry+saline+saline, mCherry+propofol+CNO, hM3D(Gq)+propofol+saline, and hM3D(Gq)+propofol+CNO groups in SNI mice at 4 h after treatment (*n* = seven mice per group). J) Total distance traveled during the OF test for mCherry+saline+saline, mCherry+propofol+CNO, hM3D(Gq)+propofol+saline, and hM3D(Gq)+propofol+CNO groups in SNI mice at 4 h after treatment (*n* = seven mice per group). Data are shown as the mean (white circles) ± SD (vertical lines) along with individual data points and were compared using one‐way ANOVA followed by Kruskal–Wallis's multiple comparisons test (C and D) or one‐way ANOVA followed by Tukey's multiple comparisons test (E, H, I and J). CFA, complete Freund's adjuvant; SNI, spared nerve injury; PVN, paraventricular nucleus; CRH, corticotropin‐releasing hormone; EPM, elevated plus maze; OF, open field; BL, baseline; CNO, Clozapine *N*‐oxide.

To further establish the causal role of PVN^CRH^ neurons in the ability of propofol to alleviate anxiety‐like behaviors, we first conducted an experiment in which propofol was locally injected into the PVN of mice subjected to CFA. We established that the local administration of propofol to the brain significantly mitigated pain‐associated anxiety‐like behaviors (Figure S10, Supporting Information). Subsequently, we injected rAAV‐hSyn‐DIO‐hM3D(Gq)‐mCherry and rAAV‐hSyn‐DIO‐mCherry bilaterally into the PVN of saline‐ and CFA mice (**Figure** **4**A,B). The perfusion of brain slices with CNO increased the firing rate of PVN^CRH^ neurons (Figure S9, Supporting Information). Chemogenetic activation of PVN^CRH^ neurons blocked the anxiety‐relieving effects of propofol in the CFA mice (Figure 4C–E). Similar results were observed in the SNI mice (Figure 4F–J). In addition to our earlier findings, these results suggest that PVN^CRH^ neurons may be the neural target of propofol in anxiety‐like behaviors associated with pain.

### 2.5 Propofol Rebalances the Synaptic Homeostasis in PVN^CRH^ Neurons Through Increased Spontaneous Inhibitory Postsynaptic Currents

**Figure 5:**
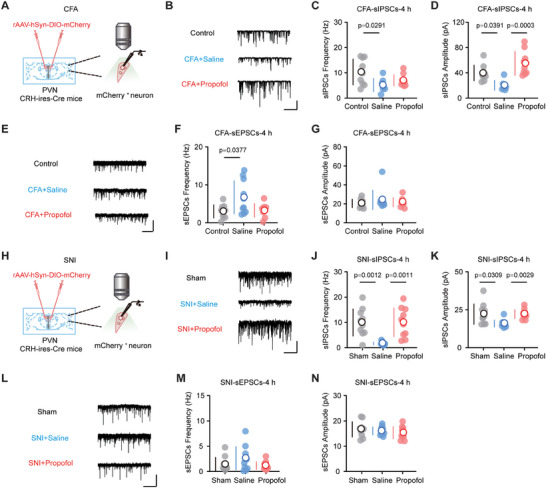
Propofol rebalances the synaptic homeostasis of PVN^CRH^ neurons by increasing inhibition. A) Schematic of whole‐cell patch‐clamp recording in the PVN brain slice from CFA mice. B) Representative traces showing sIPSCs in PVN^CRH^ neurons from an individual mouse for each group. Scale bars, 50 pA, 2.5 s. C) Average frequency of sIPSCs in different groups at 4 h after treatment (control, *n* = nine neurons in four mice; CFA + saline, *n* = seven neurons in four mice; CFA + propofol, *n* = nine neurons in four mice). D) Average amplitude of sIPSCs in different groups at 4 h after treatment (control, *n* = nine neurons in four mice; CFA + saline, *n* = seven neurons in four mice; CFA + propofol, *n* = nine neurons in four mice). E) Representative traces showing sEPSCs in PVN^CRH^ neurons from an individual mouse for each group. Scale bars, 40 pA, 2 s. F) Average frequency of sEPSCs in different groups at 4 h after treatment (control, *n* = nine neurons in four mice; CFA + saline, *n* = ten neurons in four mice; CFA + propofol, *n* = eight neurons in four mice). G) Average amplitude of sEPSCs in different groups at 4 h after treatment (control, *n* = nine neurons in four mice; CFA + saline, *n* = ten neurons in four mice; CFA + propofol, *n* = eight neurons in four mice). H) Schematic of whole‐cell patch‐clamp recording in the PVN brain slice from SNI mice. I) Representative traces showing sIPSCs in PVN^CRH^ neurons from an individual mouse for each group. Scale bars, 50 pA, 5 s. J) Average frequency of sIPSCs in different groups at 4 h after treatment (sham, *n* = ten neurons in four mice; SNI + saline, *n* = ten neurons in four mice; SNI + propofol, *n* = ten neurons in four mice). K) Average amplitude of sIPSCs in different groups at 4 h after treatment (sham, *n* = ten neurons in four mice; SNI + saline, *n* = ten neurons in four mice; SNI + propofol, *n* = ten neurons in four mice). L) Representative traces showing sEPSCs in PVN^CRH^ neurons from an individual mouse for each group. Scale bars, 50 pA, 5 s. M) Average frequency of sEPSCs in different groups at 4 h after treatment (sham, *n* = ten neurons in four mice; SNI + saline, *n* = ten neurons in four mice; SNI + propofol, *n* = ten neurons in four mice). N) Average amplitude of sEPSCs in different groups at 4 h after treatment (sham, *n* = ten neurons in four mice; SNI + saline, *n* = ten neurons in four mice; SNI + propofol, *n* = ten neurons in four mice). Data are shown as the mean (white circles) ± SD (vertical lines) along with individual data points and were compared using one‐way ANOVA followed by Tukey's multiple comparisons test (C, D, F, J, and N) or one‐way ANOVA followed by Kruskal–Wallis's multiple comparisons test (G, K, and M). CFA, complete Freund's adjuvant; SNI, spared nerve injury; PVN, paraventricular nucleus; CRH, corticotropin‐releasing hormone; CFA + Saline, CFA‐injected mice exposed to saline; CFA + Propofol, CFA‐injected mice exposed to propofol; SNI + Saline, SNI mice exposed to saline; SNI + Propofol, SNI mice exposed to propofol; sIPSCs, spontaneous inhibitory postsynaptic currents; sEPSCs, spontaneous excitatory postsynaptic currents.

To further elucidate the changes in intrinsic excitability in PVN^CRH^ neurons triggered by pain and propofol administration, we used whole‐cell patch‐clamp recordings to study how propofol affects PVN^CRH^ neurons at the cellular level (**Figure** **5**A,H).

To examine the synaptic mechanism underlying the effects of propofol in alleviating anxiety‐like behaviors during pain, we recorded spontaneous inhibitory postsynaptic currents (sIPSCs) and spontaneous excitatory postsynaptic currents (sEPSCs) in PVN^CRH^ neurons. The frequency and amplitude of the sIPSCs decreased in the CFA+saline group 4 h after treatment (Figure 5B–D). The amplitude of the sIPSCs in the CFA+propofol group was significantly higher than that in the CFA+saline group at this time point; however, their frequencies did not differ (Figure 5B–D). The frequency of sEPSCs increased in the CFA+saline group 4 h after treatment (Figure 5E,F), whereas the amplitude of sEPSCs did not differ between the control and CFA+saline groups (Figure 5E,G), and the frequency and amplitude of sEPSCs did not differ between the CFA+propofol and CFA+saline groups (Figure 5E–G).

Additionally, SNI induced a notable decrease in the amplitude and frequency of sIPSCs (Figure 5I–K), whereas sEPSCs remained unchanged, indicating weakened inhibitory synaptic transmission of PVN^CRH^ neurons (Figure 5L–N). Propofol increased the frequency and amplitude of sIPSCs (Figure 5I–K), but no alteration was observed in sEPSCs 4 h after treatment (Figure 5L–N).

Overall, these data demonstrate that propofol administration rebalances synaptic homeostasis in PVN^CRH^ neurons by increasing sIPSCs in mouse pain models.

### 2.6 Propofol Attenuates Anxiety‐Like Behaviors Associated with Pain Through GABA_A_ *β*3 Subunits in PVN^CRH^ Neurons

**Figure 6:**
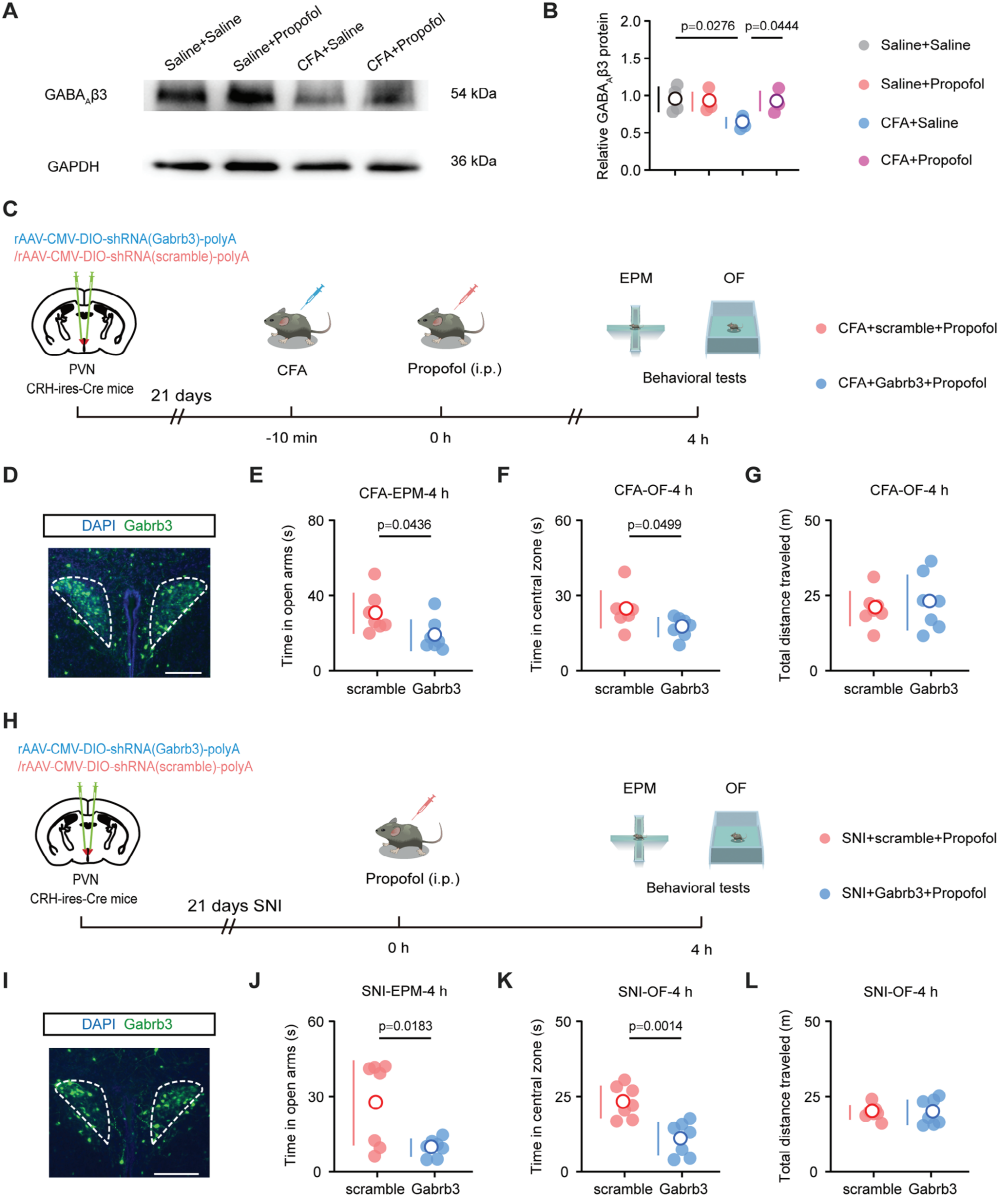
Propofol effects on and dependence on GABA_A_ *β*3 subunits in PVN^CRH^ neurons. A) Representative image of GABA_A_ *β*3 immunoblot with saline + saline, saline + propofol, CFA + saline, and CFA + propofol samples. Proteins were isolated from the PVN tissue and GAPDH served as a loading control. B) Relative expression level of GABA_A_ *β*3 in the PVN tissue from saline + saline, saline + propofol, CFA + saline, and CFA + propofol mice (*n* = four mice per group). C) Schematic of virus injection to express Gabrb3 (GABA_A_ *β*3‐specific) or scramble shRNA on PVN^CRH^ neurons from CFA mice. D) Successful expression of Gabrb3 (GABA_A_ *β*3‐specific) shRNA on PVN^CRH^ neurons from CFA mice. Scale bar, 200 µm. E) Time in the open arms during the EPM test for scramble + propofol and Gabrb3 + propofol groups in CFA mice at 4 h after treatment (*n* = seven mice per group). F) Time in the center zone during the OF test in scramble + propofol and Gabrb3 + propofol groups in CFA mice at 4 h after treatment (*n* = seven mice per group). G) Total distance traveled during the OF test in scramble + propofol and Gabrb3 + propofol groups in CFA mice at 4 h after treatment (*n* = seven mice per group). H) Schematic of virus injection to express Gabrb3 (GABA_A_ *β*3‐specific) or scramble shRNA on PVN^CRH^ neurons from SNI mice. I) Successful expression of Gabrb3 (GABA_A_ *β*3‐specific) shRNA on PVN^CRH^ neurons from SNI mice. Scale bar, 200 µm. J) Time in the open arms during the EPM test for scramble + propofol and Gabrb3 + propofol groups in SNI mice at 4 h after treatment (*n* = seven mice per group). K) Time in the center zone during the OF test in scramble + propofol and Gabrb3 + propofol groups in SNI mice at 4 h after treatment (*n* = seven mice per group). L) Total distance traveled during the OF test in scramble + propofol and Gabrb3 + propofol groups in SNI mice at 4 h after treatment (*n* = seven mice per group). Data are shown as the mean (white circles) ± SD (vertical lines) along with individual data points and were compared using one‐way ANOVA followed by Tukey's multiple comparisons test (B) or two‐tailed, unpaired Student's t‐test (E–G and J–L). CFA, complete Freund's adjuvant; SNI, spared nerve injury; PVN, paraventricular nucleus; CRH, corticotropin‐releasing hormone; EPM, elevated plus maze; OF, open field; Gabrb3, GABA_A_ *β*3, *γ*‐aminobutyric acid type A receptor *β*3 subunits; GAPDH, glyceraldehyde‐3‐phosphate dehydrogenase.

The general anesthetic effects of propofol are exerted primarily through its interaction with GABA_A_.^[^ ^3^ ^]^ Propofol can activate GABA_A_ directly or enhance its response to GABA when applied along with this neurotransmitter or other agonists.^[^ ^14^ ^]^ The *β*3 subunit of GABA_A_ is one of the important targets of propofol.^[^ ^15^ ^]^ Furthermore, pain can decrease expression of the *β*3 subunits.^[^ ^16^ ^]^ To explore the molecular targets of propofol's anxiety‐relieving effects, we performed western blotting experiments to determine the expression level of GABA_A_ *β*3 subunits in the PVN. The CFA injection resulted in a decrease in the expression level of the GABA_A_ *β*3 subunits in the PVN, which was reversed in the propofol‐treated group (**Figure** **6**A,B).

Subsequently, we performed a bilateral injection of the rAAV‐DIO‐shRNA (Gabrb3)‐GABA_A_ *β*3 vector into the PVN of CRH‐ires‐Cre mice to knock down the GABA_A_ *β*3 expression specifically in PVN^CRH^ neurons (Figure 6C,D). The downregulation of GABA_A_ *β*3 subunits expression in PVN^CRH^ neurons reduced the anxiety‐relieving effects of propofol in the CFA mice at 4 h (Figure 6E–G), 24 h, and 3 days after treatment (Figure S11, Supporting Information) as well as in the SNI mice at 4 h (Figure 6H–L), 24 h, and 3 days after treatment (Figure S12, Supporting Information). Notably, knocking down the GABA_A_ *β*3 subunits expression in PVN^CRH^ neurons did not alter mechanical hyperalgesia in naïve mice, which provides evidence that propofol has no analgesic effect in this system (Figure S13, Supporting Information). These findings suggest that the GABA_A_ *β*3 subunits are likely involved in the mechanism by which propofol provides long‐term reduction in anxiety‐like behaviors associated with pain.

## 3 Discussion

**Figure 7:**
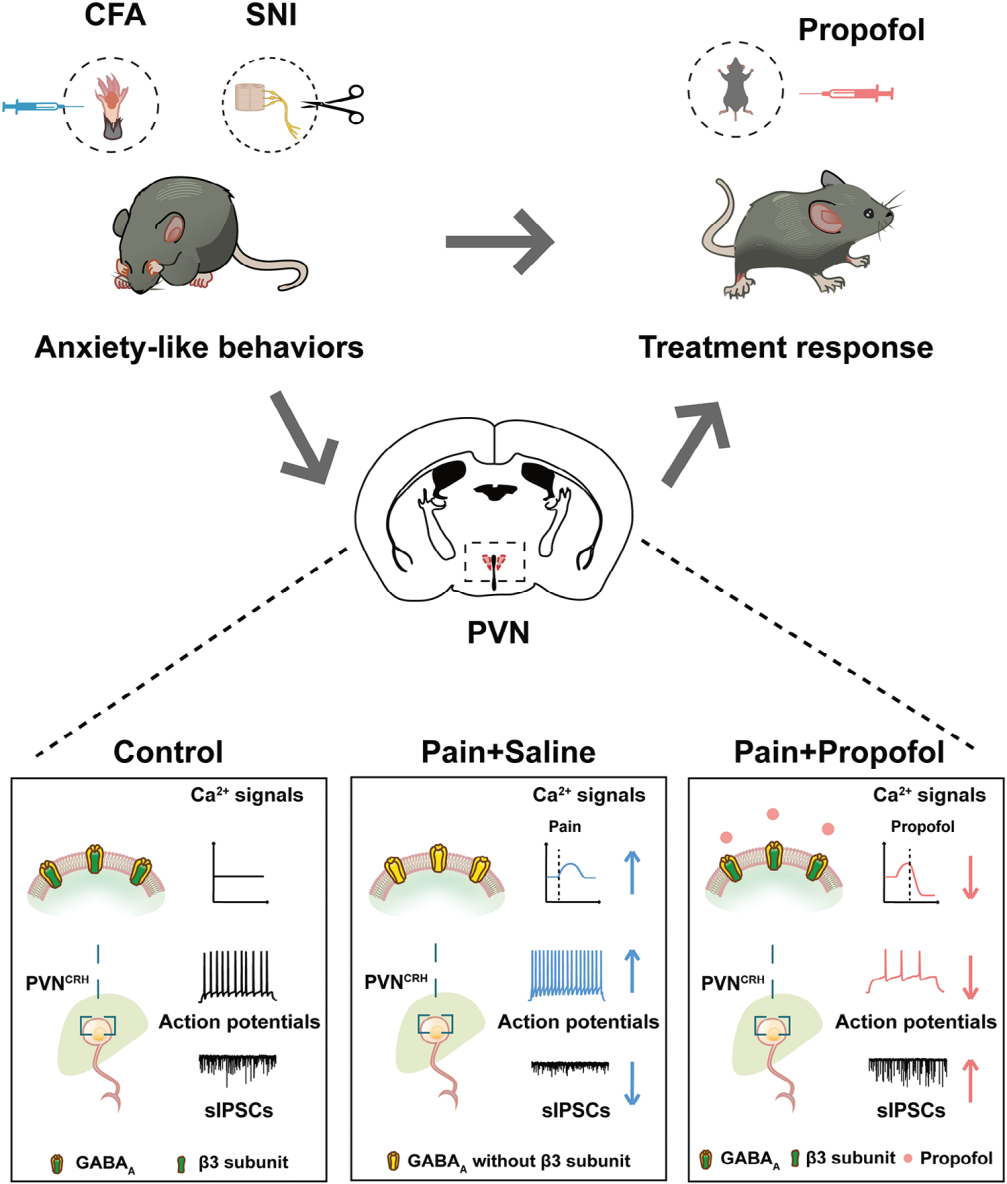
A schematic summary for how propofol alleviates anxiety‐like behaviors associated with pain by inhibiting PVN^CRH^ neurons via GABA_A_ *β*3 subunits. A single dose of propofol (100 mg kg^−1^) alleviates anxiety‐like behaviors associated with pain. This therapeutic effect is achieved by acting on GABA_A_ *β*3 subunits, thereby restoring *E*–*I* balance and subsequently inhibiting the excessive activation of PVN^CRH^ neurons in pain mice. CFA, complete Freund's adjuvant; SNI, spared nerve injury; PVN, paraventricular nucleus; CRH, corticotropin‐releasing hormone; GABA_A_ *β*3, *γ*‐aminobutyric acid types A receptor *β*3 subunit; sIPSCs, spontaneous inhibitory postsynaptic currents.

This study demonstrated that a single dose of propofol (100 mg kg^−1^) alleviates the anxiety‐like behaviors associated with pain. This therapeutic effect is achieved by acting on GABA_A_ *β*3 subunits, which restores excitation–inhibition (*E*–*I*) balance and subsequently inhibits the excessive activation of PVN^CRH^ neurons in mouse pain models (**Figure** **7**). These results provide an experimental basis for novel clinical applications of propofol and support the feasibility of targeted treatment of emotional components of pain.

### 3.1 Efficacy of Propofol as a Pharmacological Intervention for Pain‐Related Anxiety

Owing to its ability to induce euphoric moods and provide lasting relaxation, propofol has the potential to be a treatment option for mood disorders.^[^ ^4^ ^]^ We used models of acute and chronic pain to evaluate the effects of propofol on anxiety‐like behaviors associated with pain. Propofol rapidly improves anxiety‐like behaviors associated with acute and chronic pain, and this anxiolytic effect lasts for at least three days after treatment. These results indicated that propofol may be an effective pharmacological intervention for the treatment of pain‐associated anxiety.

However, we observed that anxiety levels in mice treated with propofol did not fully return to normal. This could potentially be attributed to the administration method used in our experiment, which involved intraperitoneal injections. Although we took steps to enhance the mice's adaptability through handling and intraperitoneal injection training for 5 days before the experiment, it is important to note that the injection procedure itself is invasive and can serve as a stressor, potentially elevating anxiety levels in mice. Furthermore, pain‐associated anxiety‐like behaviors are mediated by multiple brain nuclei and neural circuits,^[^ ^17^ ^]^ including the PVN, the lateral septum (LS), the lateral hypothalamus (LH), the anterior cingulate cortex (ACC), the medial prefrontal cortex (mPFC), the basolateral amygdala (BLA), and the ventral hippocampus (vHPC). Propofol may exert its effects on certain nuclei, whereas others may not respond to its anxiolytic properties. It is worth noting that the saline plantar‐injected mice used for comparison in our behavioral tests were from a different batch than the propofol‐treated mice. Mice from different batches exhibited slight variations in their baseline behavioral activities, which could contribute to the differences in anxiety levels observed between the groups.

### 3.2 PVN^CRH^ Neurons Serve as a Neural Target for the Anxiolytic Effects of Propofol

PVN^CRH^ neurons play a crucial role in integrating and regulating stress and stress‐induced negative emotions.^[^ ^18^ ^]^ In response to pain, PVN^CRH^ neurons initiate the pain response via the HPA axis neuroendocrine pathway, which ultimately leads to anxiety‐like behaviors.^[^ ^19^ ^]^ Direct activation of PVN^CRH^ neurons can cause anxiety‐like behaviors and conditioned place aversion, whereas inhibition of PVN^CRH^ neurons can alleviate both effects.^[^ ^4^, ^20^ ^]^ Thus, inhibiting the excessive activation of PVN^CRH^ neurons during pain conditions may be an effective treatment for reducing pain‐induced anxiety‐like behaviors. Indeed, our results indicate that propofol alleviates anxiety‐like behaviors associated with pain by inhibiting PVN^CRH^ neurons. Furthermore, our results revealed that local administration of propofol to the PVN significantly mitigated pain‐associated anxiety‐like behaviors. These findings support our hypothesis that PVN is a critical target for the anxiolytic effects of propofol. Given that local administration circumvents the systemic circulation and directly influences the neural substrates implicated in anxiety, this approach provides a more precise demonstration of the mechanistic action of propofol on anxiety regulation.

Our study indicates that propofol‐induced modifications in the activity of PVN^CRH^ neurons primarily affect mood alterations associated with pain without exerting a direct effect on pain perception. This finding is in line with a clinical report suggesting that propofol does not improve postoperative pain outcomes in patients.^[^ ^21^ ^]^

Given the complexity of the neuro‐pathological mechanisms underlying pain‐induced anxiety‐like behaviors, it is important to note that the PVN^CRH^ neurons are not the sole contributors. Other neuronal nuclei, including the mPFC, the dorsolateral striatum (DLS), the ACC, the LS, and the BLA, together form a complex neural network that collaboratively regulates anxiety‐like behaviors associated with pain.^[^ ^11^, ^22^ ^]^ Although these neuronal nuclei and circuits play a collective role in modulating anxiety‐like behaviors and pain thresholds, it is important to consider the possibility of their synergistic involvement in the propofol‐induced alleviation of pain‐associated anxiety‐like behaviors. Future research should focus on investigating the specific role of these additional neuronal nuclei in the anxiolytic effects of propofol.

### 3.3 GABA_A_ *β*3 Subunits Serve as Molecular Targets for the Anxiolytic Effects of Propofol

At the cellular level, an imbalance between excitatory and inhibitory signals may result in changes in neuronal excitability, which may contribute to negative emotions.^[^ ^23^ ^]^ In our study, we observed a notable decrease in the frequency and amplitude of sIPSCs and an increase in the frequency of sEPSCs in PVN^CRH^ neurons in mice exhibiting pain‐induced anxiety‐like behaviors compared to the control mice, which may have resulted from the disruption of the *E*–*I* balance that ultimately led to the hyperactivation of PVN^CRH^ neurons. Propofol administration rebalanced the *E*–*I* balance by increasing sIPSCs rather than decreasing sEPSCs to reduce the hyperactivity of PVN^CRH^ neurons. This result is consistent with previous reports indicating that clinical concentrations of propofol do not significantly affect excitatory transmission.^[^ ^24^ ^]^ We observed similar results in the chronic pain model. The frequency and amplitude of sIPSCs in PVN^CRH^ neurons were decreased in the SNI mice, whereas propofol restored these reductions. These findings are consistent with those of previous studies that have reported that the balance of excitatory and inhibitory inputs in PVN^CRH^ neurons plays an essential role in regulating anxiety disorders.^[^ ^8^, ^25^ ^]^ The amplitude of sIPSCs is strongly linked to the expression and function of postsynaptic GABA receptors. Propofol acts as a GABA_A_ agonist and directly activates GABA_A_ to potentiate its response to GABA.^[^ ^26^ ^]^ Therefore, by acting on postsynaptic GABA receptors, propofol may re‐balance the *E*–*I* balance to alleviate anxiety‐like behaviors associated with pain.

Modern transcriptomic and proteomic analyses have suggested that GABA_A_α and GABA_A_ *β* subunits are the main receptor targets of propofol.^[^ ^27^ ^]^ The α subunit of GABA_A_ plays a role in regulating pain sensation.^[^ ^28^ ^]^ As 100 mg kg^−1^ propofol did not have analgesic effects, we suggest that the *α* subunit may not be the main target of propofol's anxiety‐relieving effects. The GABA_A_ *β*3 subunit is one of the most important targets of propofol.^[^ ^27b^ ^]^ The *β*3 subunit is highly expressed in PVN^CRH^ neurons,^[^ ^16^ ^]^ and has a robust effect on anxiety‐related behaviors.^[^ ^15^, ^29^ ^]^ These previous reports are consistent with our finding that propofol reversed the decrease in GABA_A_ *β*3 subunit expression in the PVN caused by the CFA injection. Indeed, our targeted downregulation of the *β*3 subunits in PVN^CRH^ neurons through RNA interference prevented the anxiety‐relieving properties of a single dose of propofol. However, downregulation of the *β*3 subunit in PVN^CRH^ neurons did not affect the threshold of mechanical hyperalgesia in naïve mice. Based on our findings, it is reasonable to conclude that the GABA_A_ *β*3 subunit may be a molecular target for propofol's anxiety‐relieving effects, although we cannot rule out the role of other subunits of the GABA_A_ in propofol's anxiety‐relieving effects. Furthermore, the GABA_A_ *β*3 subunits exert their effects through downstream molecules and signaling pathways.^[^ ^28d^ ^]^ Defining the precise molecular mechanism through which propofol regulates the activity of PVN^CRH^ neurons by GABA_A_ *β*3 subunits to alleviate anxiety‐like behaviors will require further investigation.

This study has several limitations. First, all behavioral experiments were conducted using male mice, and we did not assess the effects of propofol on anxiety‐like behaviors in female mice. As sex hormone levels are closely related to anxiety,^[^ ^30^ ^]^ the anxiety‐relieving effects of propofol may differ between the sexes. Second, we selected the EPM and OF tests as indicators of anxiety‐like behaviors. More behavioral paradigms, such as the light/dark box test, should be explored in future studies.

In conclusion, our findings indicate that a single dose of propofol (100 mg kg^−1^) can effectively rebalance *E*–*I* balance by acting on GABA_A_ *β*3 subunits to suppress the hyperactivation of PVN^CRH^ neurons, ultimately alleviating anxiety‐like behaviors associated with pain. Our results suggest that propofol can be administered to treat other psychiatric and emotional disorders.

## 4 Experimental Section

### Animals

Adult male C57BL/6J mice (8–12 weeks old) were purchased from Shanghai Model Organisms and used to establish an inflammatory pain model and a chronic pain model for behavioral tests. CRH‐ires‐Cre (CRH‐Cre) knock‐in male mice (strain no. 012704) (Jackson Laboratory, Bar Harbor, ME, USA) were used for behavioral testing, fiber photometry recording and in vitro brain slice experiments. The cages were housed four or five mice per cage under a 12 h light/dark cycle (lights on from 7 am to 7 pm) at 22–25 °C with food and water ad libitum. All experiments were approved by the Institutional Animal Care and Use Committee of ShanghaiTech University (20221028002).

### Drugs and Inflammatory Pain Model

An inflammatory pain model was first established. CFA (25 µL; Sigma–Aldrich, St. Louis, MO, USA) was injected into the left hind paw of each mouse using an insulin syringe to induce persistent inflammatory pain.^[^ ^11^, ^31^ ^]^ The control group was injected with an equal volume of saline solution. Mice had 25, 50, or 100 mg kg^−1^ of propofol (AstraZeneca UK Limited, London, UK) injected intraperitoneally or were injected with a similar volume of intralipid (Haisco Pharmaceutical Group, Chengdu, China) to exclude the effects of the solvent.

### Spared Nerve Injury (SNI) Model

Surgery was performed under isoflurane anesthesia. The skin and muscles of the left thigh were incised to expose the sural, common peroneal, and tibial nerves. Subsequently, the nerves were explored. After exploration, non‐absorbent 6–0 chromic gut sutures were used to ligate the common peroneal and tibial nerves, which were transected, and ≈2 mm sections from the dot were removed. For the sham mice, the procedure was the same as that for the experimental group, except that the nerves were left intact.^[^ ^32^ ^]^ Twenty‐one days after the SNI surgery, the mice received an intraperitoneal injection of propofol (100 mg kg^−1^).

### Behavioral Assays

Behavioral tests were performed when mice were 8–12 weeks old. The mice were placed in a room 1 h before testing to adapt to the experimental environment.^[^ ^32a^ ^]^ Behavioral tests were performed at 4 h; 24 h; or 3 days after propofol treatment. To eliminate the impact of possible adaptation on performance, each mouse underwent behavioral tests only once, and separate cohorts were used for the different time points.

### Elevated Plus Maze (EPM) Test

The EPM apparatus consisted of two closed arms (35 cm × 6 cm) and two open arms (35 cm × 6 cm), with a central platform (6 cm × 6 cm) at the confluence. Each mouse was placed on the central platform facing an open arm and was allowed to explore the maze for 5 min. The time spent in the open arm during this 5‐minute period was recorded.^[^ ^11c^ ^]^

### Open Field (OF) Test

The OF apparatus consisted of a central square area (25 cm × 25 cm), with surrounding margins (50 cm × 50 cm × 60 cm). The mice were placed in the central zone of the OF apparatus and allowed to explore freely for 5 min while their locomotor activity was recorded.^[^ ^11c^ ^]^ The total distance traveled and time spent in the central zone were calculated using behavioral software (Jiliang Software, Shanghai, China).

### Mechanical Sensitivity

The mechanical withdrawal thresholds were measured using von Frey filaments. Mice were placed individually in a plastic chamber and allowed to acclimate for 1 h, after which they were tested for the paw withdrawal thresholds. von Frey filaments were used in ascending order to exert 0.02 to 1.4 g in the middle of the left hind paw surface. The mean thresholds were calculated for the five applications.^[^ ^11c^ ^]^

### Enzyme‐Linked Immunosorbent Assay (ELISA)

Blood samples were collected from the experimental mice (the sampling time points were consistent with the behavioral testing time points) and centrifuged at 15 g for 15 min. The resulting serum samples were promptly frozen at −80 °C until further use. Serum CORT, CRH, and NE levels were measured using ELISA Kits (Jiangsu Jingmei Biological Technology Co., Ltd., Yancheng, China).

### Stereotaxic Viral Injections

Mice were anesthetized with isoflurane and mounted on a stereotaxic instrument (RWD, ShenZhen, China).^[^ ^33^ ^]^ Each virus solution was slowly injected (30 nL min^−1^) into the PVN either unilaterally (for fiber photometry) or bilaterally (for chemogenetic experiments). The PVN coordinates relative to the bregma were –0.65 mm AP, ±0.25 mm ML, –4.67 mm DV. The glass electrode was held in place at the injection site for 10 min to allow the diffusion of the viral particles. Surgical procedures were similar to those described previously.^[^ ^8^ ^]^ Viruses (including rAAV‐hSyn‐DIO‐GCaMP7b, rAAV‐hSyn‐DIO‐hM3D(Gq)‐mCherry, rAAV‐hSyn‐DIO‐hM4D(Gi)‐mCherry, rAAV‐hSyn‐DIO‐mCherry, rAAV‐CMV‐DIO‐shRNA(Gabrb3)‐WPRE‐hGH‐polyA and rAAV‐CMV‐DIO‐shRNA(scramble)‐WPRE‐hGH‐polyA) were purchased from BrainVTA (Wuhan, China) (Figures S14 and S15, Supporting Information).

### Fiber Photometry Recording

The optical fiber (outer diameter, 200 µm; numerical aperture, 0.37; Anilab) was inserted into the ceramic ferrule and introduced into the PVN after the rAAV‐hSyn‐DIO‐GCaMP7b virus injection. Fluorescent signals were acquired using a fiber photometry system (including a 488 nm excitation laser, 505‐ to 544‐nm emission filter and a photomultiplier tube [R3896]; Hamamatsu). The signals were recorded using Spike 2 software (CED, Cambridge, UK). Photometry data were exported from Spike 2 to MATLAB R2020b Mat files for analysis. The values of fluorescence changes (*ΔF/F*) were derived by calculating (*F*−*F_0_*)/*F_0_*, where *F_0_* was the baseline fluorescence signal averaged over a 5‐s‐long control time window. The Δ*F/F* values were presented as average plots to illustrate the signal changes trial by trial. The analytical procedures were similar to those described previously.^[^ ^8^ ^]^

### Chemogenetic Inhibition of PVN^CRH^ Neurons

PVN^CRH^ neurons were bilaterally injected with rAAV‐hSyn‐DIO‐hM4D(Gi)‐mCherry or rAAV‐hSyn‐DIO‐mCherry. The mice were placed in the testing room for 1 h before the behavioral tests. Behavioral tests were performed 1 h after CNO (3 mg kg^−1^, i.p.) was injected into each mouse.

### Chemogenetic Activation of PVN^CRH^ Neurons

PVN^CRH^ neurons were bilaterally injected with rAAV‐hSyn‐DIO‐hM3D(Gq)‐mCherry or rAAV‐hSyn‐DIO‐mCherry. The mice were placed in the testing room 1 h before the behavioral tests to allow acclimation. CNO (3 mg kg^−1^, i.p.) was injected into each mouse in the treated group 30 min before propofol injection, whereas an equal volume of saline was injected into each mouse in the control group. Behavioral tests were performed 4 h after the propofol or saline injection.

### Cannula Infusion

A double guide cannula (center‐to‐center distance 0.3 mm, RWD) was placed above the PVN. After the mice had recovered for at least 14 days, propofol (10 µm) was microinjected with a double injector cannula, which had a 0.5‐mm extension beyond the tip of the guide cannula.

### Knockdown of GABA_A_ *β*3 Subunits in PVN^CRH^ Neurons

PVN^CRH^ neurons were bilaterally injected with rAAV‐CMV‐DIO‐shRNA(Gabrb3)‐WPRE‐hGH‐polyA or rAAV‐CMV‐DIO‐shRNA(scramble)‐WPRE‐hGH‐polyA. The mice were placed in the testing room for 1 h before the behavioral tests.

### Brain Slice Electrophysiology

Brain slice electrophysiology was performed as previously described.^[^ ^4a^ ^]^ Adult (6–8 weeks old) male CRH‐ires‐Cre mice were injected with rAAV‐hSyn‐DIO‐mCherry into the PVN. To induce acute pain: two weeks later, mice were injected with CFA in their left hind paws and injected intraperitoneally with propofol or saline. To induce chronic pain: the SNI surgery was performed on the mice after virus injection. Twenty‐one days later, the SNI mice were injected with saline or propofol (100 mg kg^−1^). Four hours after treatment, the mice were anaesthetized with tribromoethanol and perfused with ice‐cold oxygenated (95% O2; 5% CO2) NMDG artificial cerebrospinal fluid (ACSF) solution, which consisted of 93 mm NMDG, 93 mm HCl, 2.5 mm KCl, 1.25 mm NaH2PO4, 10 mm MgSO4•7H2O, 30 mm NaHCO3, 25 mm glucose, 20 mm HEPES, 5 mm sodium ascorbate, 3 mm sodium pyruvate, and 2 mm thiourea. After perfusion, the brain was rapidly dissected and immediately transferred to an ice‐cold oxygenated NMDG ACSF solution. Brain tissue was sectioned in the coronal plane at a thickness of 300 mm using a vibratome (VT1200 S, Leica, Wetzlar, Germany). Brain slices were incubated in oxygenated NMDG ACSF at 32 °C for 10–15 min and then transferred into normal oxygenated ACSF (126 mm NaCl, 2.5 mm KCl, 1.25 mm NaH2PO4, 2 mm MgSO4•7H2O, 10 mm glucose, 26 mm NaHCO3, and 2 mm CaCl2) at room temperature for 1 h. All chemicals used in slice preparation were purchased from Sigma–Aldrich.

Slices were transferred to the recording chamber with ACSF perfusion at a rate of 3 mL min^−1^ at 28 °C. Whole‐cell patch‐clamp recordings were made from PVN^CRH^ neurons and visualized with an Olympus BX61W1 microscope (equipped with GFP and mCherry filters) using infrared video microscopy and differential interference contrast optics. Recording pipettes (3–4 MΩ) were prepared using a micropipette puller (P2000, Sutter Instrument, USA). For whole‐cell recording, the pipettes were filled with the ACSF solution containing 133 mm potassium gluconate, 18 mm NaCl, 0.6 mm EGTA, 10 mm HEPES, 2 mm MgATP, and 0.3 mm Na3•GTP (pH, 7.2; 280 mOsm). For APs evoked by current injections, a current‐step protocol (from −25 to 200 pA, with 25–pA increments for PVN recording) was run and repeated. The neurons were held at −70 mV in voltage‐clamp mode to record sIPSCs and sEPSCs for 5 min.

### Western Blot

Total proteins were extracted from the PVN to detect the expression of GABA_A_ *β*3 subunits. RIPA buffer working solution was used to extract proteins. The proteins in each sample were quantified using a bicinchoninic acid (BCA) protein assay (Thermo Fisher Scientific, MA, USA). Equal amounts of protein (20 µg) were electrophoresed on a 10% SDS‐polyacrylamide gel followed by electrotransfer onto polyvinylidene difluoride (PVDF) membranes (Millipore, Bedford, MA, USA) (Figure S15, Supporting Information).

Membranes were blocked for 2 h in TBST (150 mm NaCl, 10 mm Tris, 0.1% Tween 20, pH 7.6) containing 10% BSA. The primary antibody against GABA_A_ *β*3 (Abcam, Cambridge, UK, ab300063–40µL) was diluted in blocking buffer and incubated overnight at 4 °C. The blots were incubated with horseradish peroxidase‐conjugated secondary antibodies for 1 h at room temperature after three washes in phosphate–buffered saline (PBS). The blots were exposed to an enhanced chemiluminescent substrate after three additional washes in PBS. Densitometric analysis of protein bands was performed by an independent blinded observer using Image J software.

### Statistical Analysis

No statistical methods were used to pre‐determine the sample sizes; however, our sample sizes were similar to those in previous reports.^[^ ^4^, ^34^ ^]^ In our experiments, three mice were excluded from data collection and analysis because of failed fiber implantation, and nine mice were excluded from data collection and analysis because of virus injection misalignment. Data were analyzed using GraphPad Prism 8.0 (GraphPad Software, San Diego, CA, USA) or MATLAB R2020b. Unpaired Student's t‐test was used for comparisons between two independent groups. Data were subjected to one‐way ANOVA followed by Tukey's multiple comparisons test if they passed the Kolmogorov–Smirnov normality test or followed by the Kruskal–Wallis's multiple comparisons test if they failed the normality test.^[^ ^35^ ^]^ Two‐way ANOVA followed by Tukey's comparison test was used for multiple comparisons.^[^ ^36^ ^]^ Data were presented in the text as the mean and standard deviation (SD) in the form “mean ± SD”. P<0.05 was considered statistically significant.

## Conflict of Interest

The authors declare no conflict of interest.

## Author Contributions

L.Y. and X.Z. contributed equally to this work. J.H. and Y.Z. designed the study. L.Y., X.Z., K.P., H.Q., K.Y., and F.C. performed the experiment. L.Y. wrote original manuscript. L.Y., X.Z., J.H., and Y.Z. revised the manuscript. J.H. and Y.Z. performed supervision. All authors reviewed and gave final approval to the manuscript.

## Supporting information

Supporting Information

## Data Availability

The data that support the findings of this study are available from the corresponding author upon reasonable request.
