# Supplementary material for: Propofol Alleviates Anxiety‐Like Behaviors Associated with Pain by Inhibiting the Hyperactivity of PVNCRH Neurons via GABAA Receptor β3 Subunits
Source: Adv Sci (Weinh). 2024 Apr 19;11(28):2309059. doi: 10.1002/advs.202309059 (PMC11267288; doi:10.1002/advs.202309059)
Supplement: Supplementary file 1 — Supporting Information [file ADVS-11-2309059-s001.pdf]

ADVANCED SCIENCE
Open Access

# Supporting Information

for *Adv. Sci.*, DOI 10.1002/advs.202309059

Propofol Alleviates Anxiety-Like Behaviors Associated with Pain by Inhibiting the Hyperactivity of $PVN^{CRH}$ Neurons via $GABA_A$ Receptor $\beta 3$ Subunits

*Le Yu, Xiaona Zhu, Kang Peng, Huimin Qin, Kexin Yang, Fang Cai, Ji Hu* and Ye Zhang**

**Propofol Alleviates Anxiety-Like Behaviors Associated with Pain by Inhibiting the Hyperactivity of $PVN^{CRH}$ Neurons via $GABA_A$ Receptor β3 Subunits**

*Le Yu, Xiaona Zhu, Kang Peng, Huimin Qin, Kexin Yang, Fang Cai, Ji Hu**, *Ye Zhang* *

L. Yu, K. Peng, Y. Zhang

Department of Anesthesiology

The Second Affiliated Hospital of Anhui Medical University

Hefei 230601, China

E-mail: zhangy@ahmu.edu.cn

L. Yu, K. Peng, Y. Zhang

Key Laboratory of Anesthesiology and Perioperative Medicine of Anhui Higher Education Institutes Anhui Medical University

Hefei 230032, China

X. Zhu, H. Qin, K. Yang, F. Cai, J. Hu

School of Life Science and Technology

ShanghaiTech University

Shanghai 201210, China

E-mail: huji@shanghaitech.edu.cn

Keywords: anxiety, corticotrophin-releasing hormone, excitatory-inhibitory balance, $GABA_A$ receptor, paraventricular nucleus, propofol

# Supporting information

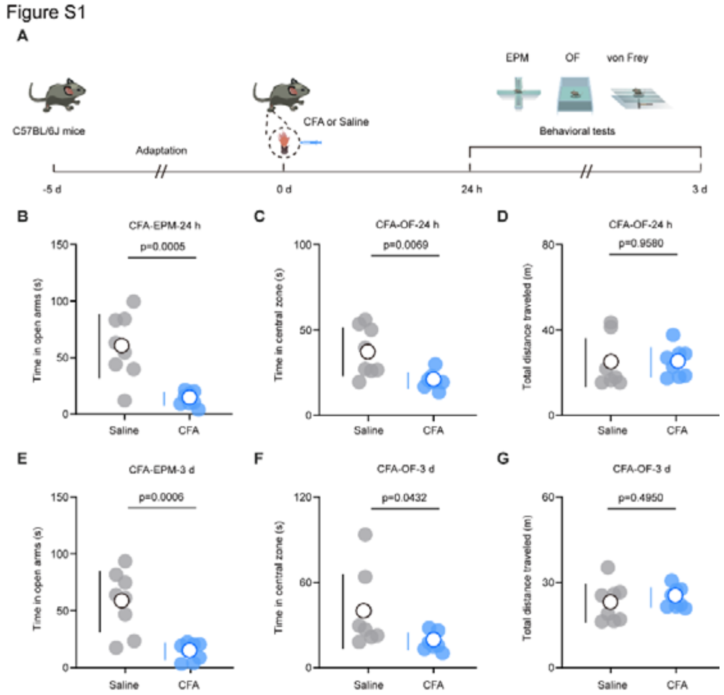


**Figure S1.** Pain induce anxiety-like behaviors at 24 h and 3 d after CFA injection. A) Schematic of the injection of CFA along with behavioral tests. B) Time in the open arms during the EPM test for saline and CFA groups at 24 h after injection (*n*=8 mice per group). C) Time in the central zone during the OF test for saline and CFA groups at 24 h after injection (*n*=8 mice per group). D) Total distance traveled during the OF test for saline and CFA groups at 24 h after injection (*n*=8 mice per group). E–G) Data as described for (B–D) but at 3 d after injection (*n*=8 mice per group). Data are shown as the mean (white circles) ± SD (vertical lines) along with individual data points and were compared using two-tailed, unpaired Student's t-test. CFA, complete Freund's adjuvant; EPM, elevated plus maze; OF, open field.

Figure S2

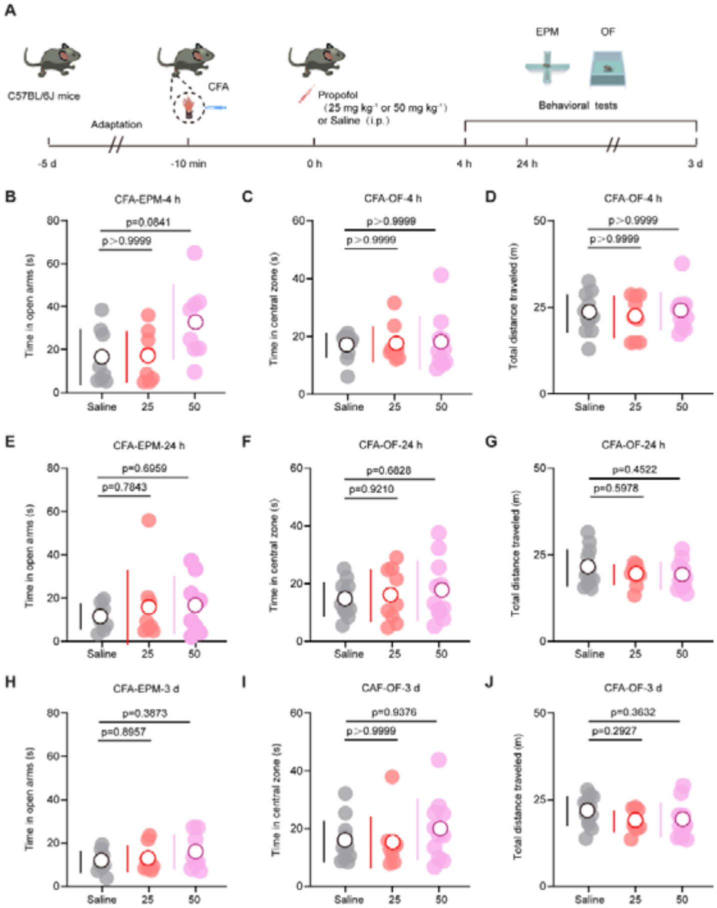


**Figure S2.** Propofol at 25 mg $kg^{-1}$ or 50 mg $kg^{-1}$ has no effect on anxiety-like behaviors in CFA mice. A) Schematic of the injection of CFA and propofol (25 mg $kg^{-1}$ or 50 mg $kg^{-1}$) along with behavioral tests. B) Time in the open arms during the EPM test for CFA + saline, CFA + propofol (25 mg $kg^{-1}$), and CFA + propofol (50 mg $kg^{-1}$) groups at 4 h after drug injection ($n$=8 mice per group). C) Time in the central zone during the OF test for CFA + saline ($n$=12 mice), CFA + propofol (25 mg $kg^{-1}$) ($n$=9 mice), and CFA + propofol (50 mg $kg^{-1}$) ($n$=11 mice) groups at 4 h after drug injection. D) Total distance traveled during the OF test for CFA + saline ($n$=12 mice), CFA + propofol (25 mg $kg^{-1}$) ($n$=9 mice), and CFA + propofol (50 mg $kg^{-1}$) ($n$=11 mice) groups at 4 h after drug injection. E–G) Data as described for (B–D) but at 24 h after CFA injection. H–J) Data as described for (B–D) but at 3 d after drug injection. Data are shown as the mean (white circles) ± SD (vertical lines) along with individual data points and were compared using one-way ANOVA followed by Tukey's multiple comparisons test (B, E–H and J) or one-way ANOVA followed by Kruskal–Wallis's multiple comparisons test (C, D and I). CFA, complete Freund's adjuvant; EPM, elevated plus maze; OF, open field; CFA + Saline, CFA-injected mice exposed to saline; CFA + Propofol, CFA-injected mice exposed to propofol.

Figure S3

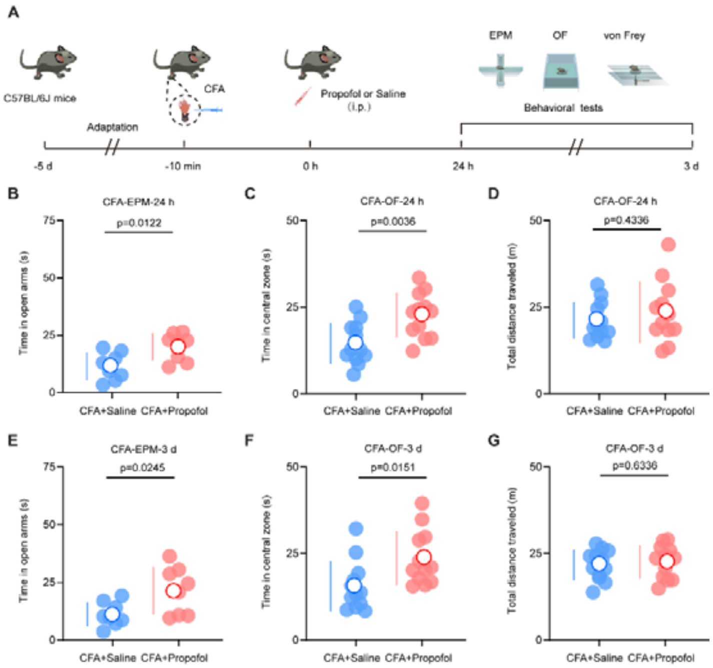


**Figure S3.** Propofol has long-lasting effects on anxiety-like behaviors in CFA mice at 24 h and 3 d. A) Schematic of the injection of CFA and propofol (100 mg $kg^{-1}$) along with behavioral tests. B) Time in the open arms during the EPM test for CFA + saline and CFA + propofol groups at 24 h after drug injection (*n*=8 mice per group). C) Time in the central zone during the OF test for CFA + saline and CFA + propofol groups at 24 h after drug injection (*n*=12 mice per group). D) Total distance traveled during the OF test for CFA + saline and CFA + propofol groups at 24 h after drug injection (*n*=12 mice per group). E–G) Data as described for (B–D) but at 3 d after drug injection. Data are shown as the mean (white circles) ± SD (vertical lines) along with individual data points and were compared using two-tailed, unpaired Student's t-test. CFA, complete Freund's adjuvant; EPM, elevated plus maze; OF, open field; CFA + Saline, CFA-injected mice exposed to saline; CFA + Propofol, CFA-injected mice exposed to propofol.

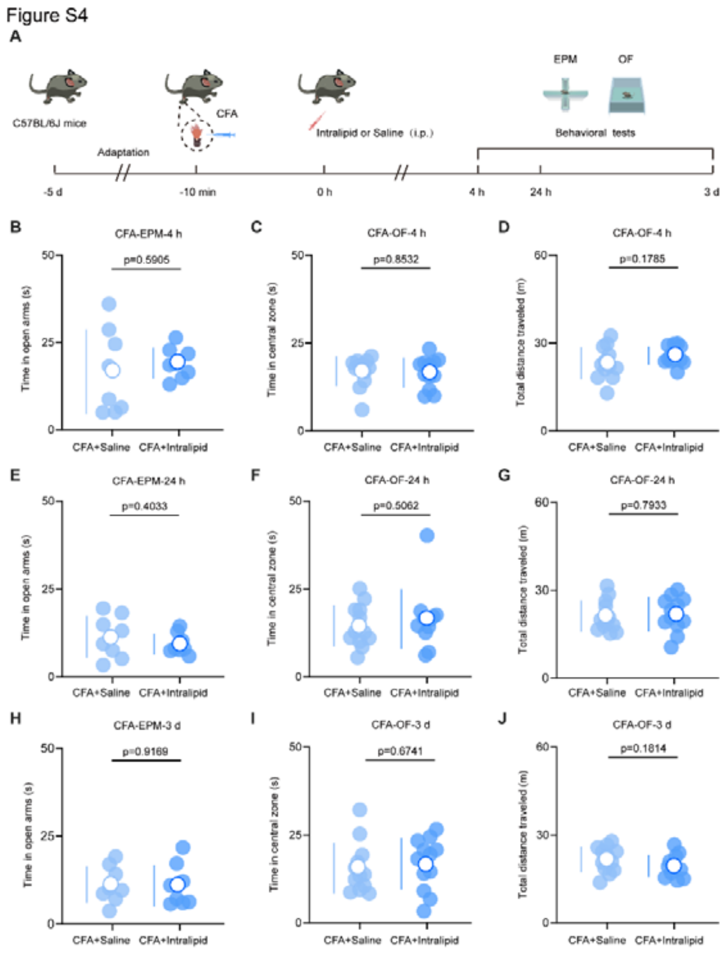


**Figure S4.** Intralipid has no effect on anxiety-like behaviors in CFA mice. A) Schematic of the injection of CFA and intralipid along with behavioral tests. B) Time in the open arms during the EPM test for CFA + saline and CFA + Intralipid groups at 4 h after drug injection (*n*=8 mice per group). C) Time in the central zone during the OF test for CFA + saline and CFA + Intralipid groups at 4 h after drug injection (*n*=12 mice per group). D) Total distance traveled during the OF test for CFA + saline and CFA + Intralipid groups at 4 h after drug injection (*n*=12 mice per group). E–G) Data as described for (B–D) but at 24 h after treatment. H–J) Data as described for (B–D) but at 3 d after treatment. Data are shown as the mean (white circles) ± SD (vertical lines) along with individual data points and were compared using two-tailed, unpaired Student's t-test. CFA, complete Freund's adjuvant; EPM, elevated plus maze; OF, open field; CFA + Saline, CFA-injected mice exposed to saline; CFA + Intralipid, CFA-injected mice exposed to Intralipid.

Figure S5

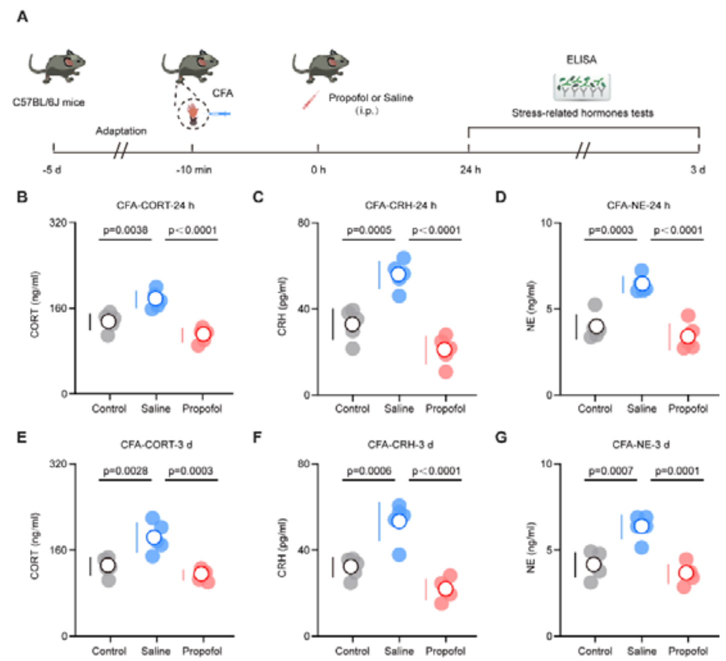


**Figure S5.** Propofol suppresses endocrine responses in CFA mice at 24 h and 3 d after treatment. A) Schematic of the injection of CFA and propofol along with stress-related hormones tests. B) Serum CORT concentrations for control, CFA + saline, and CFA + propofol groups at 24 h after drug injection (*n*=5 mice per group). C) Serum CRH concentrations for control, CFA + saline, and CFA + propofol groups at 24 h after drug injection (*n*=5 mice per group). D) Serum NE concentrations for control, CFA + saline, and CFA + propofol groups at 24 h after drug injection (*n*=5 mice per group). E–G) Data as described for (B–D) but at 3 d after drug injection (*n*=5 mice per group). Data are shown as the mean (white circles) ± SD (vertical lines) along with individual data points and were compared using one-way ANOVA followed by Tukey's multiple comparisons test. CFA, complete Freund's adjuvant; ELISA, enzyme linked immunosorbent assay; CORT, corticosterone; CRH, corticotropin-releasing hormone; NE, norepinephrine; CFA + Saline, CFA-injected mice exposed to saline; CFA + Propofol, CFA-injected mice exposed to propofol.

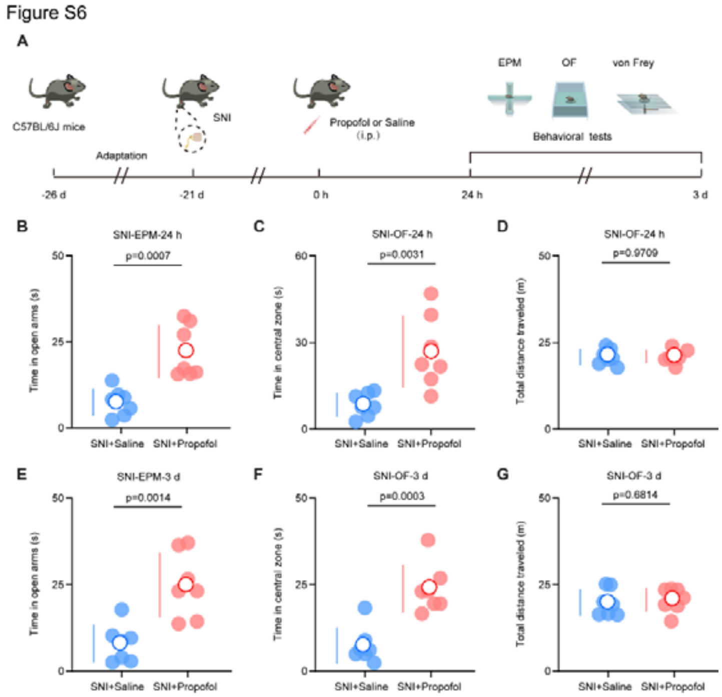


**Figure S6.** Propofol has long-lasting effects on anxiety-like behaviors in SNI mice. A) Schematic of the injection of propofol (100 mg $kg^{-1}$) along with behavioral tests in SNI mice. B) Time in the open arms during the EPM test for SNI + saline and SNI + propofol groups at 24 h after drug injection (*n*=7 mice per group). C) Time in the central zone during the OF test for SNI + saline and SNI + propofol groups at 24 h after drug injection (*n*=7 mice per group). D) Total distance traveled during the OF test for SNI + saline and SNI + propofol groups at 24 h after drug injection (*n*=7 mice per group). E–G) Data as described for (B–D) but at 3 d after drug injection (*n*=7 mice per group). Data are shown as the mean (white circles) ± SD (vertical lines) along with individual data points and were compared using two-tailed, unpaired Student's t-test. SNI, spared nerve injury; EPM, elevated plus maze; OF, open field; SNI + Saline, SNI mice exposed to saline; SNI + Propofol, SNI mice exposed to propofol.

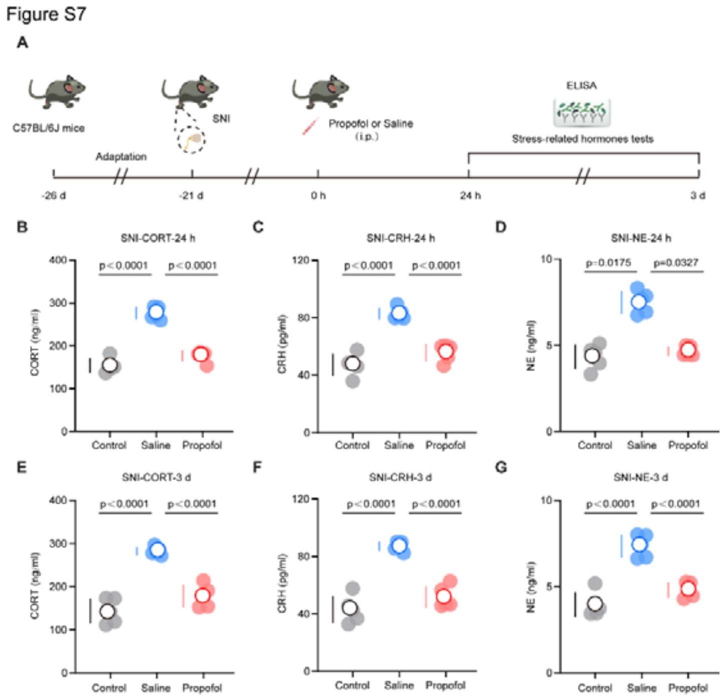


**Figure S7.** Propofol suppresses endocrine responses in SNI mice. A) Schematic of the injection of propofol along with stress-related hormones tests in SNI mice. B) Serum CORT concentrations for sham, SNI + saline, and SNI + propofol groups at 24 h after drug injection (*n*=5 mice per group). C) Serum CRH concentrations for sham, SNI + saline, and SNI + propofol groups at 24 h after drug injection (*n*=5 mice per group). D) Serum NE concentrations for sham, SNI + saline, and SNI + propofol groups at 24 h after drug injection (*n*=5 mice per group). E–G) Data as described for (B–D) but at 3 d after drug injection (*n*=5 mice per group). Data are shown as the mean (white circles) ± SD (vertical lines) along with individual data points and were compared using one-way ANOVA followed by Tukey's multiple comparisons test (B, C, E–G) or one-way ANOVA followed by Kruskal–Wallis's multiple comparisons test (D). SNI, spared nerve injury; ELISA, enzyme linked immunosorbent assay; CORT, corticosterone; CRH, corticotropin-releasing hormone; NE, norepinephrine; SNI + Saline, SNI mice exposed to saline; SNI + Propofol, SNI mice exposed to propofol.

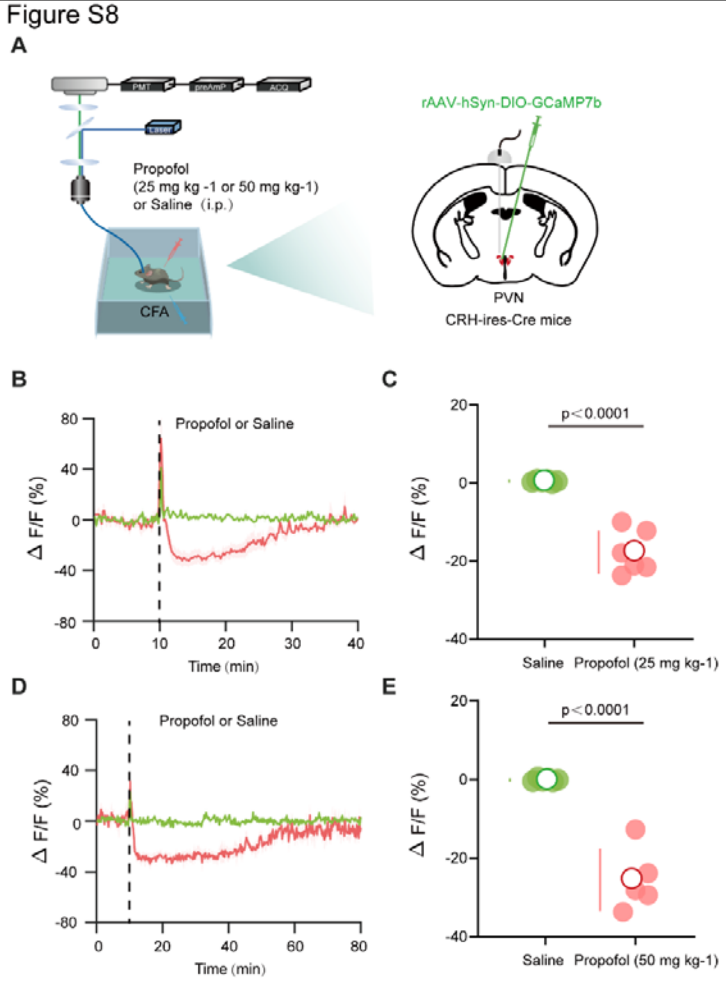


**Figure S8.** The calcium response dynamics of PVN$^{CRH}$ neurons to propofol at 25 mg kg$^{-1}$ or 50 mg kg$^{-1}$. A) Schematic of fiber implantation above PVN$^{CRH}$ neurons expressing GCaMP7b in CRH-ires-Cre mice. B) GCaMP7b signals from PVN$^{CRH}$ neurons relative to the moment of the administration of saline and 25 mg kg$^{-1}$ propofol (*n*=6 mice per group). C) Quantification of the changes in GCaMP7b signals after administration of saline and 25 mg kg$^{-1}$ propofol. D) GCaMP7b signals from PVN$^{CRH}$ neurons relative to the moment of the administration of saline (*n*=6 mice) and 50 mg kg$^{-1}$ propofol (*n*=5 mice). E) Quantification of the changes in GCaMP7b signals after administration of saline and 50 mg kg$^{-1}$ propofol. Data are shown as the mean (white circles) ± SD (vertical lines) along with individual data points and were compared using two-tailed, unpaired Student's t-test. CFA, complete Freund's adjuvant; PVN, paraventricular nucleus; CRH, corticotropin-releasing hormone.

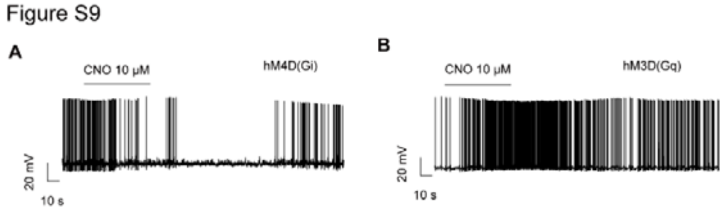


**Figure S9.** Chemical genetic modulation of PVN$^{CRH}$ neuronal excitability. A) Representative traces of whole-cell current-clamp recordings showing that CNO inhibits action potential firing in hM4D(Gi)-expressing PVN$^{CRH}$ neurons. Scale bars, 20 mV, 10 s. B)

Representative traces of whole-cell current-clamp recordings showing that CNO activates action potential firing in hM3D(Gq)-expressing PVN$^{CRH}$ neurons. Scale bars, 20 mV, 10 s.

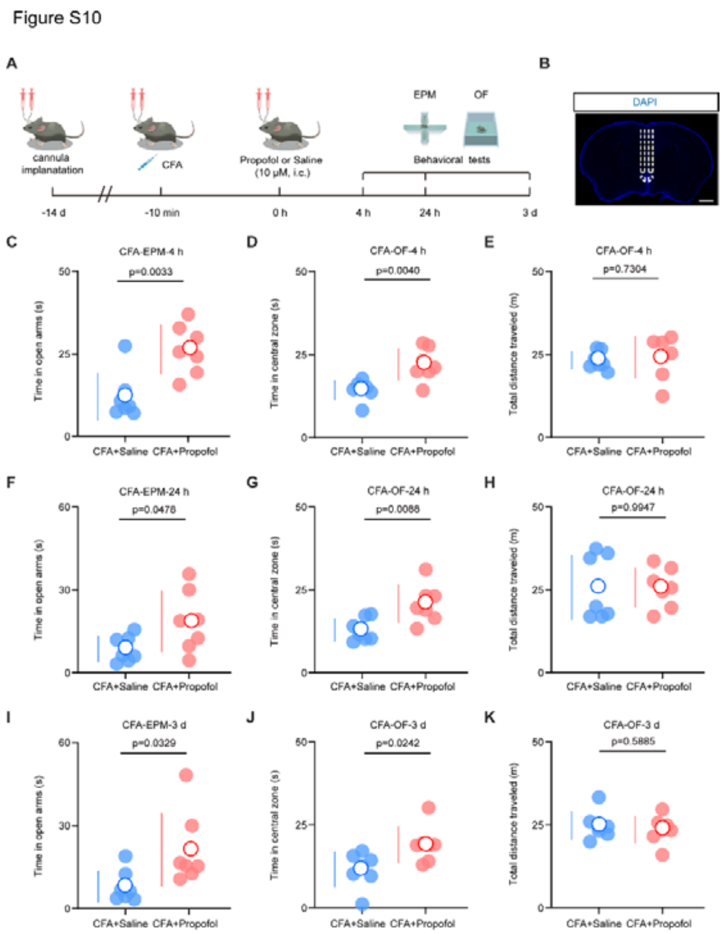


**Figure S10.** Local injection of propofol alleviates anxiety-like behaviors in CFA mice. A) Schematic of the injection of CFA and propofol along with behavioral tests. B) Histological verification of bilateral implantation of cannulae in mice. Scale bar, 200 µm. C) Time in the open arms during the EPM test for CFA + saline and CFA + propofol groups at 4 h after drug injection (*n*=7 mice per group). D) Time in the central zone during the OF test for CFA + saline and CFA + propofol groups at 4 h after drug injection (*n*=7 mice per group). E) Total distance traveled during the OF test for CFA + saline and CFA + propofol groups at 4 h after drug injection (*n*=7 mice per group). F–H) Data as described for (C–E) but at 24 h after drug injection (*n*=7 mice per group). I–K) Data as described for (C–E) but at 3 d after drug injection (*n*=7 mice per group). Data are shown as the mean (white circles) ± SD (vertical lines) along with individual data points and were compared using two-tailed, unpaired Student's t-test. CFA, complete Freund's adjuvant; EPM, elevated plus maze; OF, open field.

Figure S11

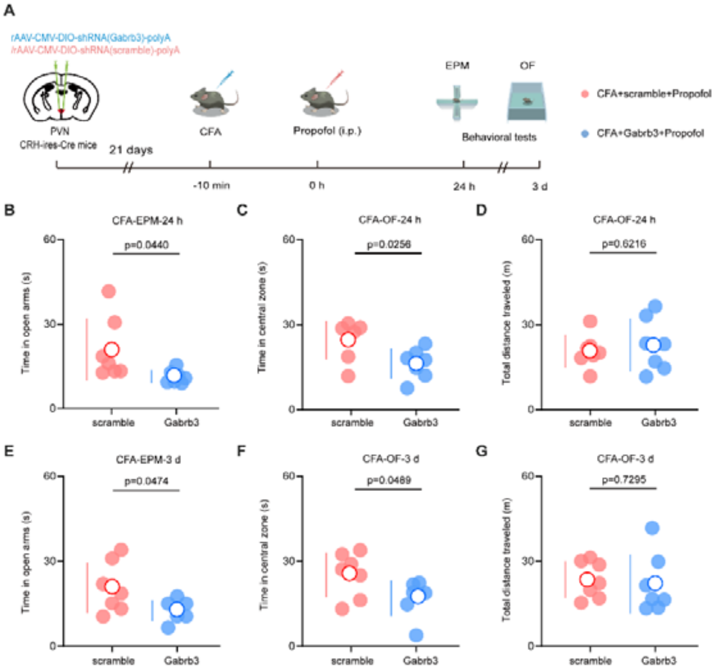


**Figure S11.** Knockdown of GABA$_A$β3 subunits in PVN$^{CRH}$ neurons abolishes the effect of propofol in CFA mice. A) Schematic of virus injection to express Gabrb3 (GABA$_A$β3-specific) or scramble shRNA on PVN$^{CRH}$ neurons in CFA mice. B) Time in the open arms during the EPM test for scramble + propofol and Gabrb3 + propofol groups at 24 h after drug injection (*n*=7 mice per group). C) Time in the central zone during the OF test for scramble + propofol and Gabrb3 + propofol groups at 24 h after drug injection (*n*=7 mice per group). D) Total distance traveled during the OF test for scramble + propofol and Gabrb3 + propofol groups at 24 h after drug injection (*n*=7 mice per group). E–G) Data as described for (B–D) but at 3 d after drug injection (*n*=7 mice per group). Data are shown as the mean (white circles) ± SD (vertical lines) along with individual data points and were compared using two-tailed, unpaired Student's t-test. EPM, elevated plus maze; OF, open field; Gabrb3, γ-aminobutyric acid type A receptor β3 subunits.

Figure S12

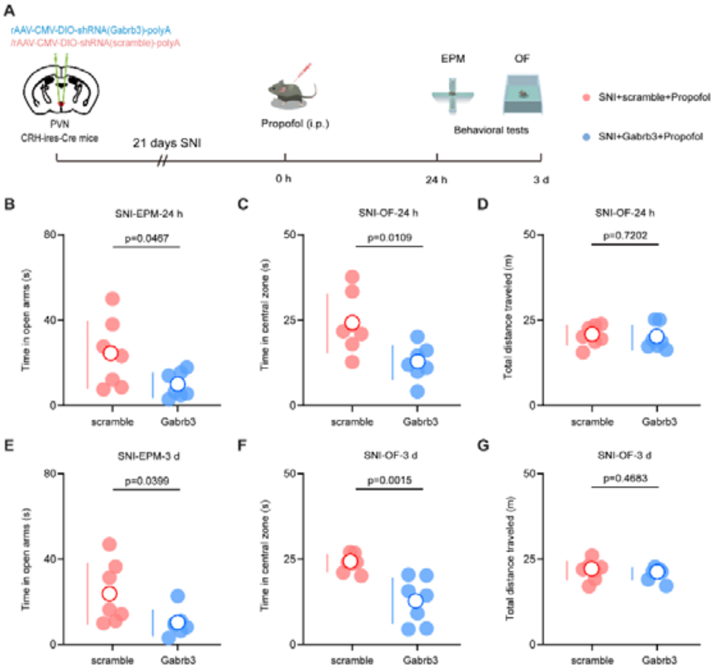


**Figure S12.** Knockdown of GABA$_A$β3 subunits in PVN$^{CRH}$ neurons abolishes the effect of propofol in SNI mice. A) Schematic of virus injection to express Gabrb3 (GABA$_A$β3-specific) or scramble shRNA on PVN$^{CRH}$ neurons in SNI mice. B) Time in the open arms during the EPM test for scramble + propofol and Gabrb3 + propofol groups at 24 h after drug injection (*n*=7 mice per group). C) Time in the central zone during the OF test for scramble + propofol and Gabrb3 + propofol groups at 24 h after drug injection (*n*=7 mice per group). D) Total distance traveled during the OF test for scramble + propofol and Gabrb3 + propofol groups at 24 h after drug injection (*n*=7 mice per group). E–G) Data as described for (B–D) but at 3 d after drug injection (*n*=7 mice per group). Data are shown as the mean (white circles) ± SD (vertical lines) along with individual data points and were compared using two-tailed, unpaired Student's t-test. EPM, elevated plus maze; OF, open field; Gabrb3, γ-aminobutyric acid types A receptor β3 subunits.

Figure S13

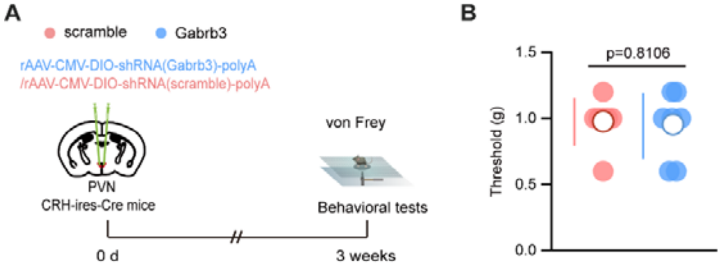


**Figure S13.** Knockdown of GABA$_A$β3 subunits in PVN$^{CRH}$ neurons has no effect on sensory hypersensitivity in naïve mice. A) Timeline of the virus injection and behavioral tests. B) Paw withdrawal threshold in the scramble and Gabrb3 groups (*n*=7 mice per group). Data are shown as the mean (white circles) ± SD (vertical lines) along with individual data points and were compared using two-tailed, unpaired Student's t-test. Gabrb3, γ-aminobutyric acid types A receptor β3 subunits.

Figure S14

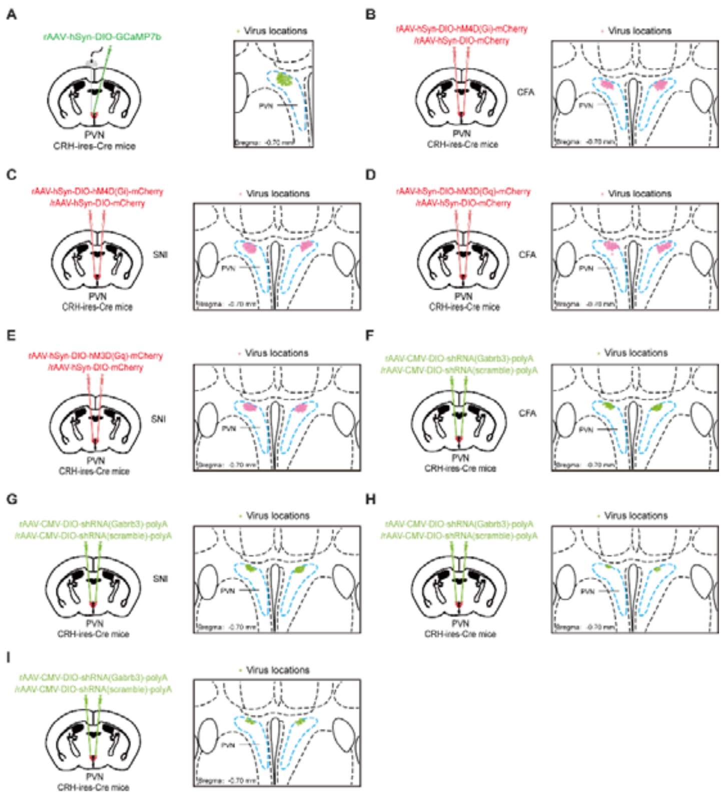


**Figure S14.** Images showing the virus injection site. A) Injection locations of DIO-GCaMP7b in CRH-ires-Cre mice. B) Injection locations of DIO-hM4D(Gi) and DIO-mCherry in CFA mice. C) Injection locations of DIO-hM4D(Gi) and DIO-mCherry in SNI mice. D) Injection locations of DIO-hM3D(Gq) and DIO-mCherry in CFA mice. E) Injection locations of DIO-hM3D(Gq) and DIO-mCherry in SNI mice. F) Injection locations of DIO-shRNA(Gabrb3) and DIO-shRNA(scramble) in CFA mice. G) Injection locations of DIO-shRNA(Gabrb3) and DIO-shRNA(scramble) in SNI mice. H) Injection locations of DIO-shRNA(Gabrb3) and DIO-shRNA(scramble) for testing knockdown efficiency. I) Injection locations of DIO-shRNA(Gabrb3) and DIO-shRNA(scramble) for testing mechanical hypersensitivity. CFA, complete Freund's adjuvant; SNI, spared nerve injury; PVN, paraventricular nucleus; CRH, corticotropin-releasing hormone.

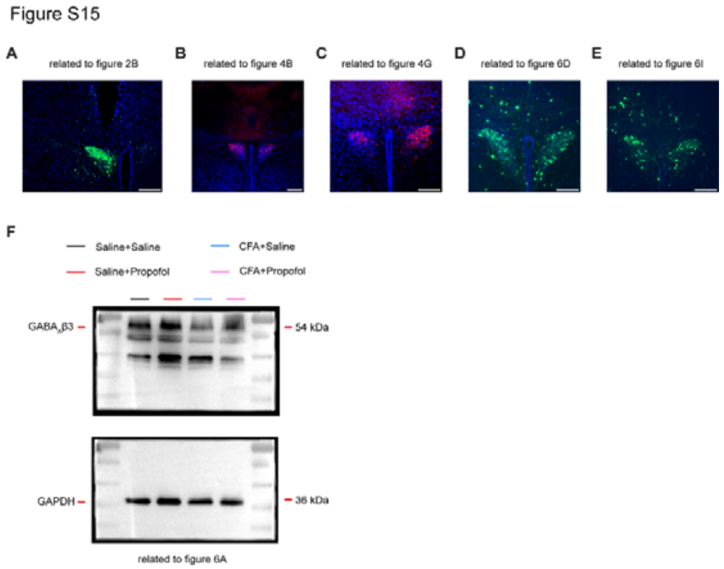


**Figure S15.** Raw data of exemplary unilateral and bilateral injections and the western blot data for the $GABA_A\beta3$ subunit assays. A) Histological verification of DIO-GCaMP7b injection and optical fiber implantation in CRH-ires-Cre mice, related to figure 2B. Scale bar, 200 µm. B) Histological verification of DIO-hM3D(Gq) injection in CRH-ires-Cre mice, related to figure 4B. Scale bar, 200 µm. C) Histological verification of DIO-hM3D(Gq) injection in CRH-ires-Cre mice, related to figure 4G. Scale bar, 200 µm. D) Histological verification of DIO-shRNA(Gabrb3) injection in CRH-ires-Cre mice, related to figure 6D. Scale bar, 200 µm. E) Histological verification of DIO-shRNA(Gabrb3) injection in CRH-ires-Cre mice, related to figure 6I. Scale bar, 200 µm. F) $GABA_A\beta3$ subunits protein levels in PVN, related to figure 6A. CFA, complete Freund's adjuvant; $GABA_A\beta3$, γ-aminobutyric acid type A receptor β3 subunits; GAPDH, glyceraldehyde-3-phosphate dehydrogenase.
